# The Tolman-Eichenbaum Machine: Unifying Space and Relational Memory through Generalization in the Hippocampal Formation

**DOI:** 10.1016/j.cell.2020.10.024

**Published:** 2020-11-25

**Authors:** James C.R. Whittington, Timothy H. Muller, Shirley Mark, Guifen Chen, Caswell Barry, Neil Burgess, Timothy E.J. Behrens

**Affiliations:** 1 Wellcome Centre for Integrative Neuroimaging, University of Oxford, Oxford OX3 9DU, UK; 2 Institute of Neurology, UCL, London WC1N 3BG, UK; 3 Wellcome Centre for Human Neuroimaging, UCL, London WC1N 3AR, UK; 4 Institute of Cognitive Neuroscience, UCL, London WC1N 3AZ, UK; 5 School of Biological and Chemical Sciences, QMUL, London E1 4NS, UK; 6 Sainsbury Wellcome Centre for Neural Circuits and Behaviour, UCL, London W1T 4JG, UK; 7 Research department of Cell and Developmental Biology, UCL, London WC1E 6BT, UK

**Keywords:** hippocampus, entorhinal cortex, generalization, grid cells, place cells, neural networks, non-spatial reasoning, representation learning

## Abstract

The hippocampal-entorhinal system is important for spatial and relational memory tasks. We formally link these domains, provide a mechanistic understanding of the hippocampal role in generalization, and offer unifying principles underlying many entorhinal and hippocampal cell types. We propose medial entorhinal cells form a basis describing structural knowledge, and hippocampal cells link this basis with sensory representations. Adopting these principles, we introduce the Tolman-Eichenbaum machine (TEM). After learning, TEM entorhinal cells display diverse properties resembling apparently bespoke spatial responses, such as grid, band, border, and object-vector cells. TEM hippocampal cells include place and landmark cells that remap between environments. Crucially, TEM also aligns with empirically recorded representations in complex non-spatial tasks. TEM also generates predictions that hippocampal remapping is not random as previously believed; rather, structural knowledge is preserved across environments. We confirm this structural transfer over remapping in simultaneously recorded place and grid cells.

## Highlights

• Common principles for space and relational memory in the hippocampal formation
• Explains hippocampal generalization in both spatial and non-spatial problems
• Accounts for many reported hippocampal and entorhinal cell types from such tasks
• Predicts how hippocampus remaps in both spatial and non-spatial tasks

## Graphical Abstract

**Figure:**
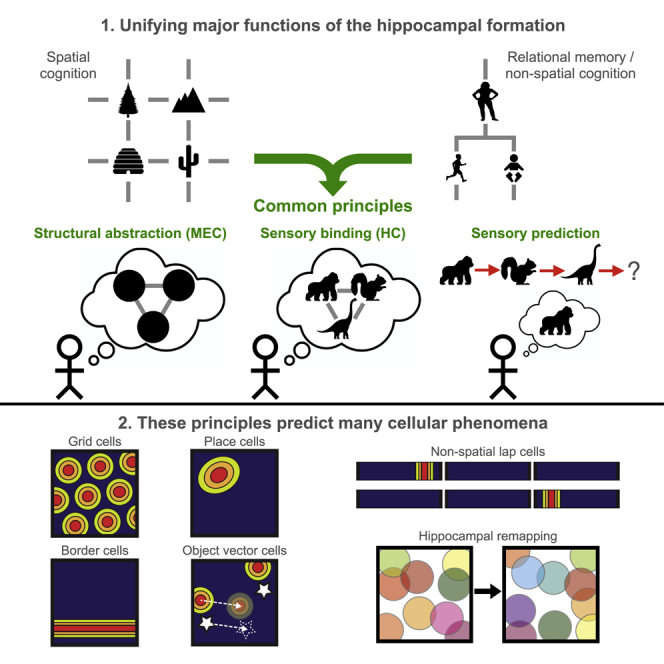

## Teaser

The Tolman-Eichenbaum Machine, named in honor of Edward Chace Tolman and Howard Eichenbaum for their contributions to cognitive theory, provides a unifying framework for the hippocampal role in spatial and nonspatial generalization and unifying principles underlying many entorhinal and hippocampal cell types.

## Introduction

Humans and other animals make complex inferences from sparse observations and rapidly integrate new knowledge to control their behavior. Tolman (1948) argued that these facilities rely on a systematic organization of knowledge called a cognitive map. In the hippocampal formation, during spatial tasks, individual neurons appear precisely tuned to bespoke features of this mapping problem (O’Keefe and Nadel, 1978; Taube et al., 1990; Hafting et al., 2005). However, the hippocampus is also critical for non-spatial inferences that rely on understanding the relationships or associations between objects and events termed relational memory (Cohen and Eichenbaum, 1993). While it has been suggested that relational memory and spatial reasoning might be related by a common mechanism (Eichenbaum and Cohen, 2014), it remains unclear whether such a mechanism exists or how it could account for the diverse array of apparently bespoke spatial cell types.

One promising approach casts spatial and non-spatial problems as a connected graph, with neural responses as efficient representations of this graph (Gustafson and Daw, 2011; Stachenfeld et al., 2017). This has led to new potential interpretations for place cells (Stachenfeld et al., 2017) and grid cells (Stachenfeld et al., 2017; Dordek et al., 2016). However, such approaches cannot account for the rapid inferences and generalizations characteristic of hippocampal function in both spatial and relational memory and do not explain the myriad types of spatial representations observed or predict how they will change across different environments (remapping).

**Figure 1:**
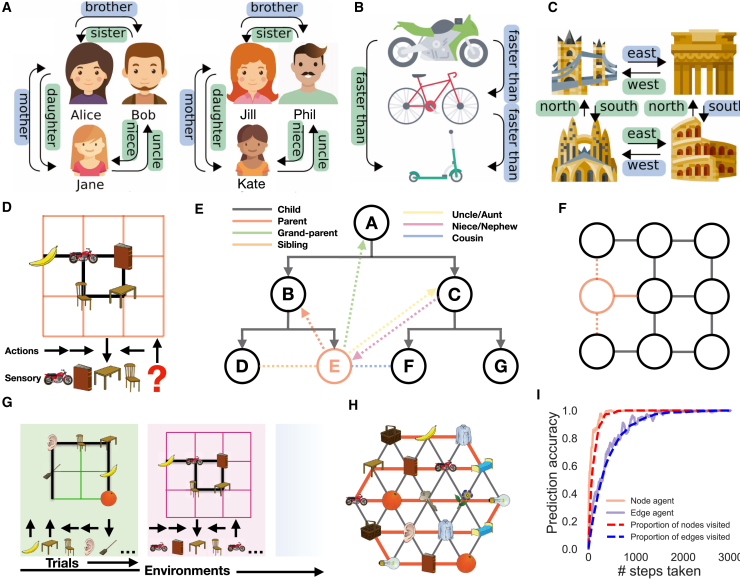
Spatial and Relational Inferences Cast as Structural Generalization (A–C) Structured relationships exist in many situations and can often be formalized on a connected graph, e.g., (A) social hierarchies, (B) transitive inference, and (C) spatial reasoning. Often the same relationships generalize across different sets of sensory objects (e.g., left/right in A). This transferable structure allows quick inference, e.g., seeing only the blue relationships allows you to infer the green ones. (D) Our task is predicting the next sensory observation in sequences derived from probabilistic transitions on a graph. Each node has an arbitrary sensory experience, e.g., a banana. An agent transitions on the graph observing only the immediate sensory stimuli and associated action taken, e.g., having seen motorbike → book → table → chair, it should predict the motorbike next if it understands the rules of the graph. (E) If you know the underlying structure of social hierarchies, observing a new node (in red) via a single relationship, e.g., Emily is Bob’s daughter, allows immediate inference about the new node’s (Emily’s) relationship to all other nodes (shown in black/gray). (F) Similarly for spatial graphs observing a new node on the left (solid red line) also tells us whether it is above or below (dashed red lines) other surrounding nodes. (G) Our agent performs this next step prediction task in many worlds sharing the same underlying structure (e.g., 6- or 4-connected graphs), but differing in size and arrangement of sensory stimuli. The aim is to learn the common structure in order to generalize and perform quick inferences. (H) Knowing the structure allows full graph understanding after only visiting all nodes, not all edges. Here, only 18 steps (red line) are required to infer all 42 links. (I) An agent that knows structure (node agent) will reach peak predictive performance after it has visited all nodes, quicker than one that has to see all transitions (edge agent). Icons from https://www.flaticon.com. See also Figure S1.

**Figure S1:**
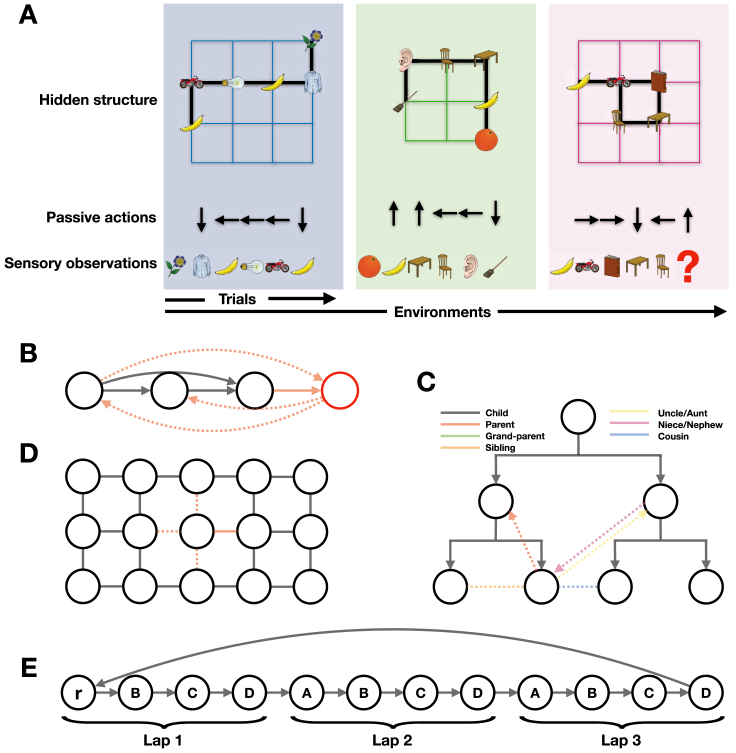
Task Schematics for TEM, Related to Figures 1 and 3 **(A)** Learning to predict the next sensory observation in environments that share the same structure but differ in their sensory observations. TEM only sees the sensory observations and associated action taken, it is not told about the underlying structure - this must be learned. (**B**) Transitive inference graph. When a new node (red) is seen to be one higher, all other (dotted) relations can be inferred i.e., 3 higher. (**C**) Example graph for a social hierarchy. (**D**) Example graph for 2D structure. (**E**) A complex task embedded in a spatial world. This is a schematic representation of the state space for the task in Sun et al. (2020). Each lap is of length 4 as the sensory objects (A, B, C, D) repeat every 4 nodes. There are 3 laps in total, and that defines the true state-space as a reward, r, is given every 3 laps.

We aim to account for this broad set of hippocampal properties by re-casting both spatial and relational memory problems as examples of structural abstraction (Kemp and Tenenbaum, 2008) and generalization (Figures 1A–1C and S1). Spatial reasoning can be cast as structural generalization, as different spatial environments share the common regularities of Euclidean space that define which inferences can be made, and which shortcuts might exist. For example, moving south→east→north→west will return you to where you started. Structural regularities also permit inferences in non-spatial relational problems. For example, transitive inference problems (which depend on the hippocampus [Bunsey and Eichenbaum, 1996; Dusek and Eichenbaum, 1997]) require stimuli to be represented on an abstract ordered line, such that A>B and B>C implies A>C. Similarly, abstraction of hierarchical structure permits rapid inferences when encountering new social situations.

Structural generalization offers dramatic benefits for new learning and flexible inference and is a key issue in artificial intelligence. One promising approach is to maintain “factorized” representations in which different aspects of knowledge are represented separately and can then be flexibly re-combined to represent novel experiences (Higgins et al., 2017). Factorizing the relationships between experiences from the content of each experience could offer a powerful mechanism for generalizing this structural knowledge to new situations. Notably, exactly such a factorization exists between sensory and spatial representations in lateral (LEC) and medial (MEC) entorhinal cortices, respectively (Manns and Eichenbaum, 2006). Manns and Eichenbaum (2006) propose that novel conjunctions of these two representations form the hippocampal representation for relational memory.

We demonstrate that this factorization and conjunction approach is sufficient to build a relational memory system (the Tolman-Eichenbaum machine [TEM]) that generalizes structural knowledge in space and non-space, predicts a broad range of neuronal representations observed in spatial and relational memory tasks, and accounts for observed remapping phenomena in both the hippocampus and entorhinal cortex. Notably, although hippocampal remapping is thought to be random, TEM predicts that this apparent randomness hides a structural representation that is preserved across environments. We verify this prediction in simultaneously recorded place and grid cells and show that suggested differences between spatial and non-spatial hippocampal remapping can be explained by this same mechanism. These results suggest a general framework for hippocampal-entorhinal representation, inference, and generalization across spatial and non-spatial tasks.

## Results

### Spatial and Relational Inferences Can Be Cast as Structural Generalization

We consider the unsupervised learning problem where an agent must predict the next sensory experience in a sequence derived from probabilistic transitions on graphs (Figure 1D). The agent does not see the graph, only a sequence of sensory observations and the “relation” or “action” that caused each transition (a transition is a jump between adjacent nodes of the graph). Different types of relation exist, e.g., in a family hierarchy, parent, aunt, child, and nephew imply different transitions on the graph, but each transition-type has the same meaning at every point on the graph. Similarly, in space, action is defined by heading direction (e.g., NESW on 4-connected graphs).

If all transitions have been experienced, the graph can be stored in memory and perfect predictions made without any structural abstraction. However, if structural properties of the graph are known *a priori*, perfect prediction is possible long before all transitions have been experienced; it only requires each node to have been experienced (Figures 1H and 1I). This can be easily understood; when the structure of the graph is known, a new node can be introduced with a single relation (Bob has a daughter, Emily; Figure 1E) and all other relations can immediately be inferred (Emily is Alice’s granddaughter and Cat’s niece, etc.). Similarly, in space, if the structure of 2D graphs is known, then placing a new node on an X-Y coordinate is sufficient to infer relational information to every other point on the graph (Figure 1F).

In summary, after experiencing many graphs with different sensory observations and learning their common relational structure, the goal of our unsupervised learning agent is to maximize its ability to predict the next sensory observation after each transition on a new graph (Figure 1G).

### The Tolman-Eichenbaum Machine

To build a machine that solves this problem, we first consider a normative solution. This is formalized as a generative model and its approximate Bayesian inversion, described in the STAR Methods. Here, we describe the key elements of this solution and their proposed mapping onto the functional anatomy of the hippocampal system.

**Figure 2:**
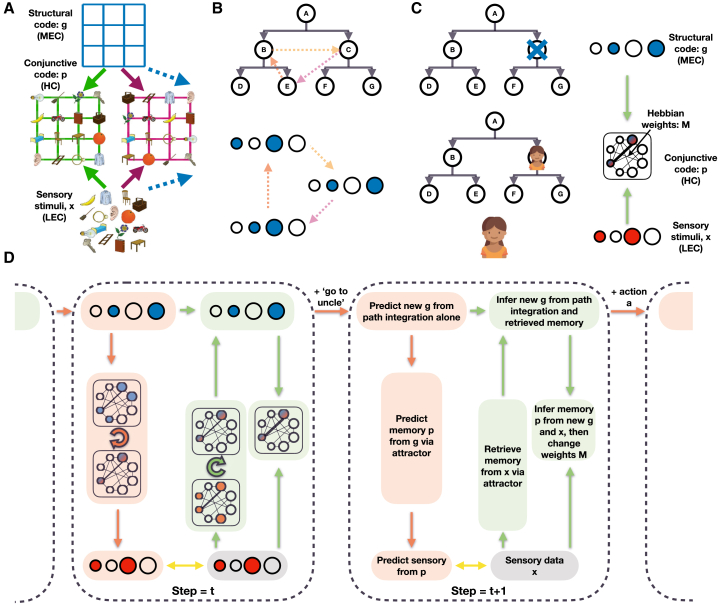
The Tolman-Eichenbaum Machine (A) Factorization and conjunction as a principle for generalization. Separating structural codes (the transition rules of the graph) from sensory codes allows generalization over environments sharing the same structure. The conjunctive code represents the current environment in the context of this learned structure. (B and C) The two key elements of TEM. (B) Representations for path integration (g) on arbitrary graphs and C) relational memories (p) that bind abstract locations to sensory observations. (B) TEM must learn structural codes (g) that (1) represent each state differently so that different memories can be stored and retrieved and (2) have the same code on returning to a state (from any direction) so the appropriate memory can be retrieved. (C) Relational memories conjunctively combine the factorized structural (in blue representing location C) and sensory (in red representing the person) codes, thus these memories know what was where. The memories are stored in Hebbian weights (M) between the neurons of p. (D) Depiction of TEM at two time points, with each time point described at a different level of detail. Red shows predictions; green shows inference. Time point *t* shows network implementation and t+1 describes each computation in words. Circles depict neurons (blue is g, red is x, blue/red is p); shaded boxes depict computation steps; arrows show learnable weights; looped arrows describe recurrent attractor. Black lines between neurons in the attractor describe Hebbian weights M. Yellow arrows show errors that are minimized during training. Overall, TEM transitions through latent variables g and stores and retrieves memories p using Hebbian weights M. We note that this is a didactic schematic; for completeness and a faithful interpretation of the Bayesian underpinnings, please see STAR Methods and Figures S2, S3, and S4.

We want to estimate the probability of the next sensory observation given all previous observations on this and all other graphs. A parsimonious solution will reflect the fact that each task is composed of two factors, a graph-structure and sensory observations (Figure 2A).

If you know the relational structure, you can know where you are even when taking paths that have not been previously experienced—a form of path integration but for arbitrary graphs (Figure 2B). Knowing where you are though is not enough for successful predictions—you also need to remember what you have seen and where you saw it. Such relational memories bind sensory observations to locations in the relational structure (Figure 2C). With these two components, sensory prediction becomes easy—path integration tells you where you are and relational memories tell you what’s there.

If these components are separated, generalization is also easy; each world has the same underlying relational structure but with a different configuration of sensory observations, thus understanding a new world is simply a problem of relational memory. More formally, to facilitate generalization of knowledge across domains we separate variables of abstract location that generalize across maps (g, general, grid cells) from those that are grounded in sensory experience and therefore specific to a particular map (p, particular, place cells).

Although p and g are variables, they are each represented as a population (vector) of units in a neural network. The problem is therefore reduced to learning neural network weights (W) that know how to (1) represent locations in relational structures (g) and (2) form relational memories (p), store them (M), and later retrieve them. Although the weights of the network are learned, we are able to make critical choices in its architecture. The resulting network maps simply onto the functional anatomy of the hippocampal formation and its computations and can be intuitively represented in schematics (Figure 2D).

### TEM and the Hippocampal Formation

Following Manns and Eichenbaum (2006), hippocampal representations, (p), are a conjunction between sensory input (x) in the LEC and abstract location (g) in the MEC. By mirroring hippocampal synaptic potentiation (Bliss and Collingridge, 1993), memories are able to be rapidly stored in weights (M) between p using simple Hebbian learning between co-active neurons and retrieved by the natural attractor dynamics of the resultant auto-associative network (Figure 2D).

To infer a new g representation, TEM performs path integration from the previous g, conditional on the current action/relation. This can be related to recurrent neural network models (RNNs) of place and grid cells (Zhang, 1996; Burak and Fiete, 2009). Like these models, different recurrent weights mediate the effects of different actions/relations in changing the activity pattern in the network (Figure 2D). Unlike these models, however, weights are learnt from sensory experience, allowing map-like abstractions and path integration to extend to arbitrary non-spatial problems.

Path integration accumulates errors (Mittelstaedt and Mittelstaedt, 1980). To overcome this problem, TEM can take advantage of a second source of information about g, the conjunctive representations, p, stored in the hippocampal memory M. TEM indexes M with the current sensory experience, x, to retrieve a set of candidate representations of g (previously visited places with a similar sensory experience) and uses these to refine the path integrated g.

When representing tasks that have self-repeating structure, it is efficient to organize cognitive maps hierarchically. To allow such hierarchy to emerge, we separate our model into multiple parallel streams, each as described above (i.e., each stream receives x, each stream’s g can transition via path integration and each stream’s p is a conjunction between its g and x [x is first temporally filtered independently for each stream; see STAR Methods]). These streams are only combined when forming and retrieving memories. When forming memories, connections, M, are also updated between active cells across streams in the hippocampus. When memories are retrieved, these same connections induce an attractor to retrieve p (see STAR Methods for details).

### Model Training

**Figure S2:**
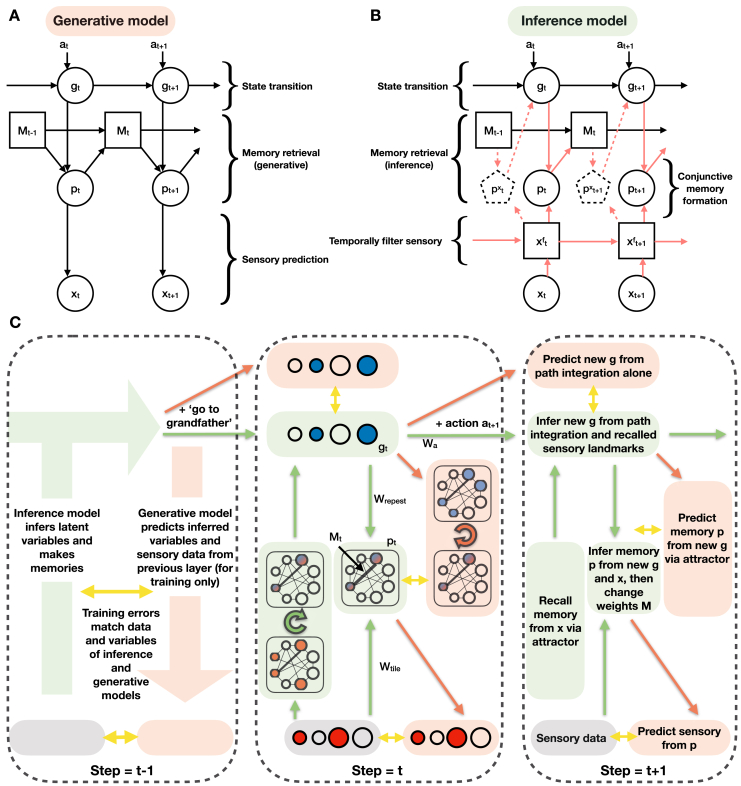
Full TEM Model, Related to Figure 2 **(A)** Generative model. (**B**) Inference model. Circled/ boxed variables are stochastic/ deterministic. Dashed arrows/boxes are optional as explained in the text. (**C**) Schematic to show the model flow in the neural network. Depiction of TEM at three time-points, with each time-point described at a different level of detail. Green/ red show inference and generative networks. Time point t−1 shows the overall Bayesian logic, *t* shows network implementation, t+1 describes each computation in words. Circles depict neurons (blue is g, red is x, blue/red is p); shaded boxes depict computation steps; arrows show learnable weights (green and red are weights in inference and generative networks); looped arrows describe recurrent attractor. Black lines between neurons in attractor describe Hebbian weights M. $W_{a}$ are learnable, action dependent, transition weights. $W_{repeat}$ and $W_{tile}$ are fixed weights that make the dimensions of the structural (blue) and sensory (red) inputs, respectively, to the attractor the same. Yellow arrows show training errors. We note we do not show the temporal filtering of sensory data x in this schematic.

**Figure S3:**
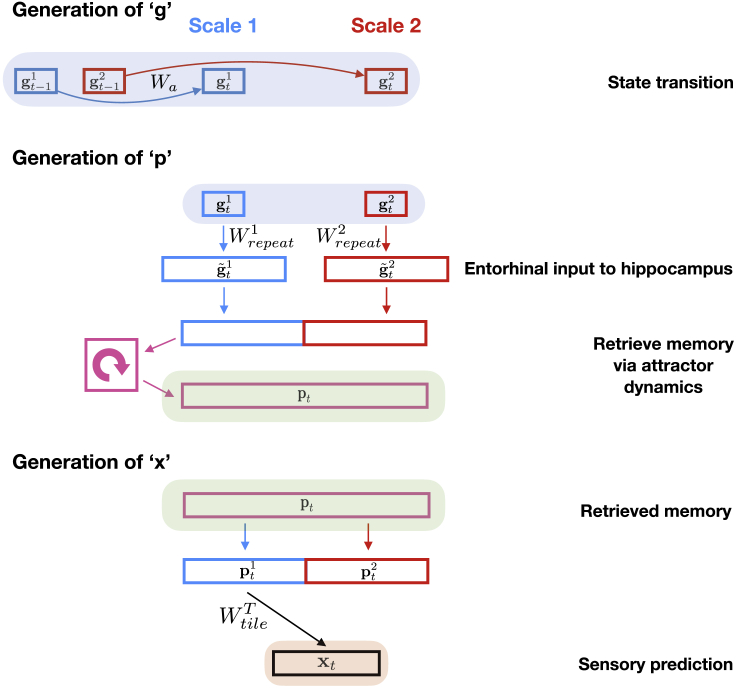
Computations in TEM Generative Model, Related to Figure 2 This shows each computation, as described in Generative architecture, making clear the fixed $W_{tile}$ and $W_{repeat}$ matrices perform appropriate dimension changes, though we note that the matrices may not be the sole computation in each step. Attractor dynamics are described in Retrieval using an attractor network. Red/Blue boxes describe two different ‘streams’.

**Figure S4:**
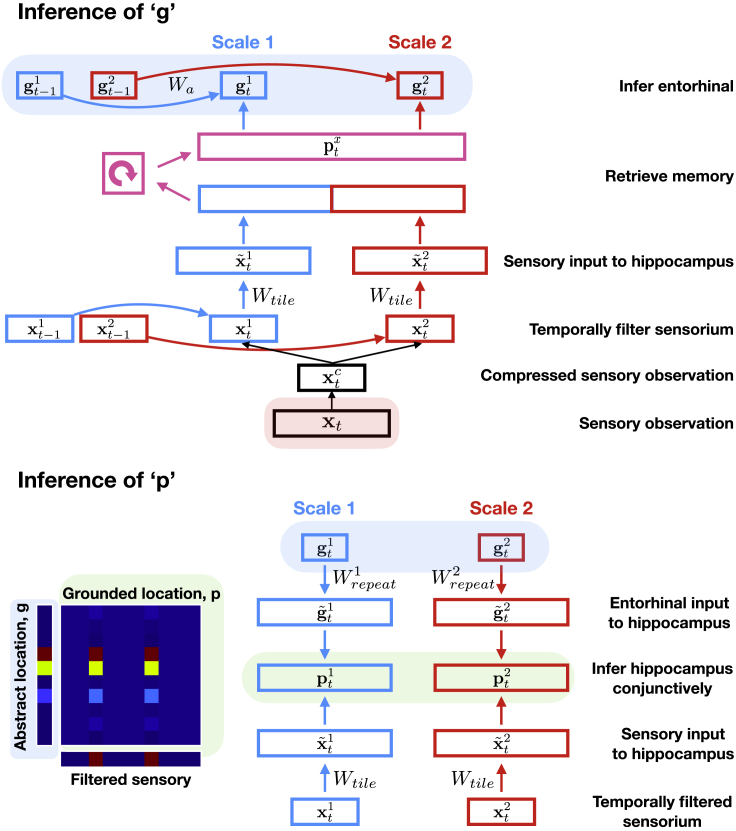
Computations in TEM Inference Model, Related to Figure 2 This shows each computation, as described in Inference architecture, making clear the fixed $W_{tile}$ and $W_{repeat}$ matrices perform appropriate dimension changes ensuring the entorhinal and sensory input to hippocampus have the same dimension. We note that the matrices may not be the sole computation in each step. Red/Blue boxes describe two different ‘streams’. In the bottom left, we show that multiplying together the representation after the $W_{tile}$ and $W_{repeat}$ operations is equivalent to an outer product.

The model’s sensory predictions are compared to sensory observations to provide an error signal. The network weights (W) are adjusted along a gradient that reduces these errors using backpropagation. In the artificial neural network model, network weights (W) differ from Hebbian weights (M). Network weights learn slowly, via backpropagation, to generalize across environments. Hebbian weights learn quickly, via Hebbian learning at every time step, to remember what is where in each environment. For aficionados, although this section describes the key elements, TEM can be framed as a generative model of graphs. This allows us to use modern Bayesian methods (Kingma and Welling, 2013; Gemici et al., 2017) to learn the network weights and perform inference on g and p. The full algorithm is detailed in the STAR Methods (Figures S2, S3, and S4).

The model is trained in multiple different environments, differing in size and sensory experience. Different environments use the same network weights, W, for path integration, but different Hebbian weights, M, for memories. The most important weights are those that transition g as they encode the structure of the map. They must ensure (1) that each location in the map has a different g representation (so a unique memory can be built) and (2) that arriving at the same location after different actions causes the same g representation (so the same memory can be retrieved)—a form of path integration for arbitrary graph structures. For example, the relation “uncle” must cause the same change in g as father followed by brother but different from brother followed by father.

To summarize, the network *only* sees sensory inputs x and the actions a at each time step. It must construct an internal representation of the environment. The g representations are a RNN; its recurrent weights define the learnt internal structure and each g representation (at a given time step) corresponding to a position in the map. When the RNN receives an action, it changes its representation, g. The aim is to make the RNN have the same representation on returning to the same point (path integration), so it can retrieve the correct memory, p. To do this, the network must implicitly learn the structure/rules of the environment. p binds a particular g representation to a particular sensory representation, x, and is stored for future recall in a set of weights, M. These weights are Hebbian and therefore change with every experience, whereas the RNN weights are fixed when the network is run and adjusted by backpropagation to minimize overall errors.

### TEM Generalizes Structural Knowledge to Novel Sensory Environments

**Figure 3:**
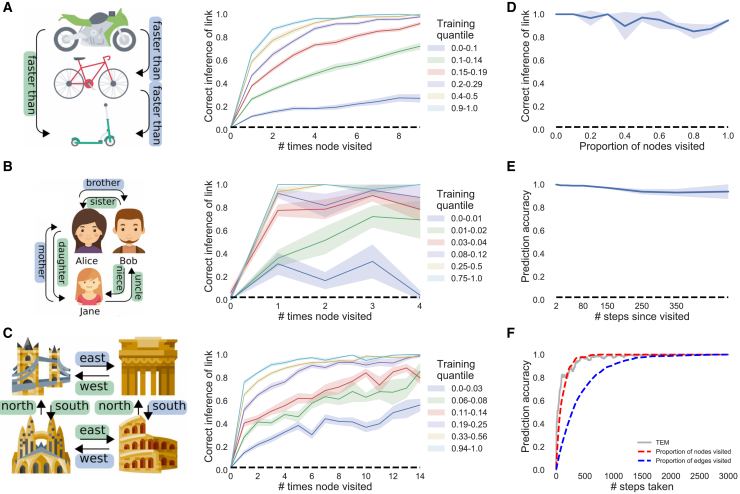
TEM Learns and Generalizes Abstract Relational Knowledge (A–C) Learning to learn: when TEM has only seen a few environments (blue/green) it takes many visits to each node to remember it. This is because it (1) does not yet understand the structure of the graph and (2) has not learned how to use memories. After visiting more environments and learning the common structure (cyan/yellow), TEM correctly predicts a node on the second visit regardless of the edge taken—TEM now understands both the rules of the graph (path integration) and how to store and retrieve memories. (A) Transitive inference, (B) social hierarchies, and (C) 2D graphs. (D–F) On 2D graphs. (D) TEM is able to predict sensory observations when returning to a node for the first time via a *new* direction—this is only possible with learned structural knowledge. (E) TEM can store long-term memories. (F) TEM’s performance tracks nodes visited, not edges. These results all demonstrate that TEM has learned and generalized abstract structural knowledge. See also Figure S1.

We first test TEM on classic non-spatial relational memory tasks thought to depend on the hippocampus—transitive inference and social hierarchy tasks (Dusek and Eichenbaum, 1997; Kumaran et al., 2012). After training, TEM immediately makes inferences in new transitive inference tasks without any additional experience (Figure 3A) e.g., after being shown sequences such as A>B>C>D>E—regardless of particular sensory identities (e.g., A,B,C,D,E or cat,dog,elephant,fox,badger) - TEM returns “B” to the query “what is 3 more than E” (the query is an action a). Therefore, TEM has learned ordinal structural knowledge. Equally, in social hierarchy tasks, TEM can infer relationships that it has never seen (Figure 3B). For example, after being shown that “Bob is the brother of Cat who is Fran’s mother”, TEM answers “Fran” when queried “who is Bob’s niece?”. In both cases, TEM was able to answer correctly without having previously seen the particular sensory details of the task before as it had been exposed to similar relational structures from which it could learn from and generalize. Such first presentation inferences are *only* possible with learned structural knowledge.

Knowing the underlying structure allows one to know the entire relational structure after a single visit to each state (Figures 1H and 1I). TEM demonstrates this data efficiency with its performance in line with the proportion of states visited in the graph, not the edges taken (Figures 3D and 3F).

We also tested TEM on tasks with an underlying spatial structure (e.g., Figures 1F and 1H). Again, TEM performed first presentation inferences in spatial generalization tasks (Figure 3C)—only possible with both learned structural knowledge and long-term relational memories (Figure 3E).

### TEM Represents Structure with Grid Cells that Generalize across Environments

**Figure 4:**
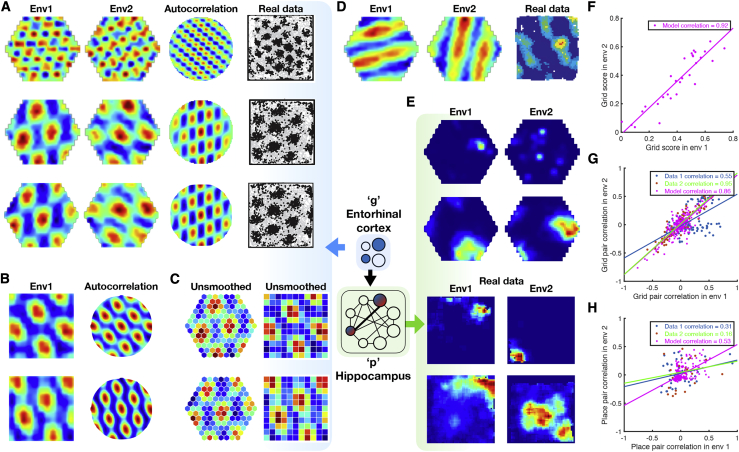
TEM Structural Neurons g Learn to Be Grid Cells that Generalize and TEM Conjunctive Memory Neurons p Learn to Be Place Cells that Remap We use 2D graphs with the number of nodes sampled from {61, 91, 127} or {64, 81, 100, 121} for hexagonal or square environments respectively. A cell’s rate map is obtained by allowing the agent to explore the environment then calculating its average firing rate at each point (graph node) in the environment. (A and B) TEM learned structural representations for random walks on 2D graphs. (A) Hexagonal worlds. Left to right: environments 1, 2, autocorrelation, real data (Krupic et al., 2012; Stensola et al., 2012), top to bottom: different cells. TEM learns grid-like cells, of different frequencies (top versus middle), and of different phases (middle versus bottom). (B) Square worlds. Two TEM learned structural cells—left/right; rate map/autocorrelation. (C) Raw unsmoothed rate maps. Left/right: bottom two cells from (A) both cells from (B). (D) TEM also learn band-like cells. Importantly, all TEM structural representations (A)–(D) generalize across environments. (E) Learned memory representations resemble place cells (left/right: environments 1/2; top 2 simulated, bottom 2 real cells) and have different field sizes. These cells remap between environments, i.e., do not generalize. (F) Grid scores of TEM grid-like cells correlate across environments. (G and H) To examine whether relationships between cells are preserved between environments, we correlated the spatial correlation coefficients of pairs of grid or place fields from each environment, using data from TEM or Barry et al. (2012) and Chen et al. (2018). (G) The spatial correlation coefficients of pairs of TEM structural cells and real data grid cells correlate strongly. (H) TEM hippocampal and real data place cells preserved their relationship to a lesser extent. This suggests that TEM structural cells, along with real grid cells, encode generalizable relationships to a greater extent than TEM hippocampal and real place cells. See also Figure S5.

**Figure S5:**
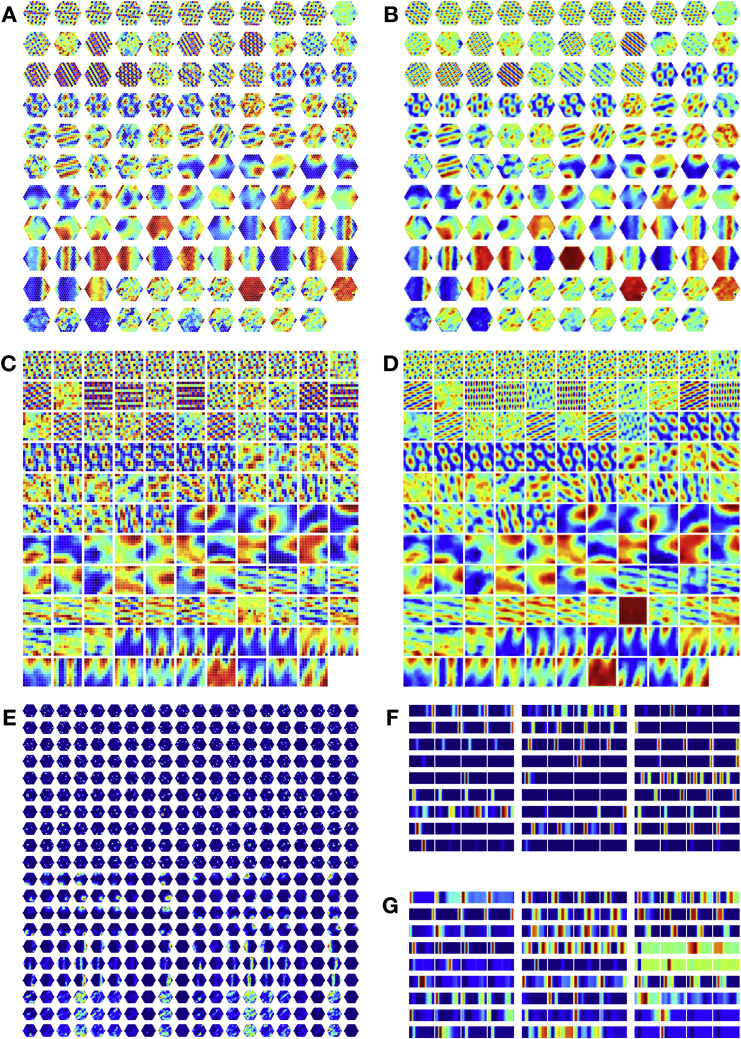
Further TEM Cell Representations, Related to Figures 4 and 7 **(A/B)** Raw/smoothed structural cells, g, learned by TEM in diffusive behavior. **C/D)** Raw/smoothed learned TEM entorhinal cells, g, when trained on a square graph environment. **D) E)** Hippocampal cells, p, learned by TEM during diffusive behavior. (**F**) Random sample of TEM hippocampal cells when trained on 4-lap task of Sun et al. (2020). (**G**) Random sample of TEM entorhinal cells when trained on 4-lap task of Sun et al. (2020).

We now interrogate the network’s learned representations to understand how they relate to the computations required for these tasks, as well as known properties of the hippocampus and entorhinal cortex. We begin by considering TEM agents that diffuse randomly on 2D graphs, constrained only by the neighborhood transitions in the environment. Here, TEM’s “abstract location” (g) representations resemble grid cells (Figures 4A and 4B for hexagonal and square environments, respectively; Figures S5A–S5D for further cells) and band cells (Figure 4D) as recorded in rodent MEC (Hafting et al., 2005; Krupic et al., 2012; Banino et al., 2018; Cueva and Wei, 2018). As in the brain, we observe modules of grid cells at different spatial frequencies and, within module, we observe cells at different grid phases (Figure 4A).

TEM’s top-level (g) representations reflect the need to be both maximally different at different spatial locations, to enable independent memories at each location, and invariant to approaching the same location from different trajectories (path integration) so that the correct memory can be retrieved. Our results suggest that these two constraints are sufficient to produce grid- and band-like representations.

Importantly, top-layer TEM representations generalize, retaining their properties across different environments. This is true of both the first- and second-order properties of the population. For example, a grid cell in environment 1 is also a grid cell of the same frequency in environment 2, and the correlation structure across grid cells is preserved—grid cells (in the same module) that fire next to each other in one environment do so in all environments. This is agnostic to environment size, thus TEM has not learned to just represent single environments but has instead learned a general representation of 2D space. These preserved properties provide the substrate for generalization of relational structure and are also observed in rodent grid cell populations recorded in multiple environments (Fyhn et al., 2007; Yoon et al., 2013).

### TEM Forms Memories with Place Cell Representations that Remap across Environments

In TEM, “hippocampal” cells, p, are a conjunction between TEM structural “medial entorhinal” cells, g, and sensory input, x; each hippocampal cell will only be active when both the structural cells and sensory input are both active (Figure S4). When purely diffusing around worlds, TEM learns sparse representations that resemble hippocampal place cells (Figures 4E and S5E). These place-like fields span multiple sizes, mirroring the hierarchical composition of hippocampal place fields (Jung et al., 1994; Kjelstrup et al., 2008).

Importantly TEM’s hippocampal cells, unlike their medial entorhinal counterparts, do not generalize. Although each environment shares the same structure, the sensory objects are distributed differently. The conjunctive nature of the hippocampal representation means that TEM’s hippocampal cells do not fully preserve their correlation structure across environments (Figure 4H) but instead relocate apparently at random in different environments. This phenomenon is commonly observed in rodent hippocampal cells and is termed *global remapping* (Anderson and Jeffery, 2003; Bostock et al., 1991; Muller and Kubie, 1987).

### Diverse Entorhinal and Hippocampal Cell Types form a Basis for Transition Statistics

**Figure 5:**
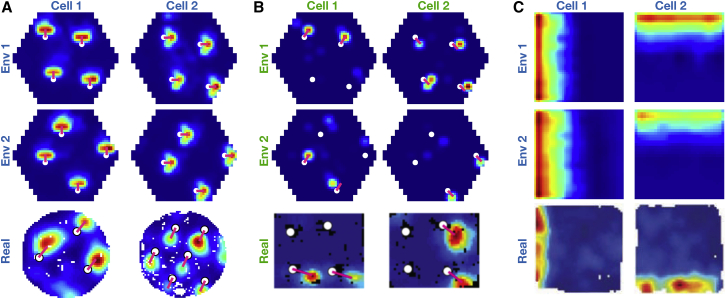
TEM Learned Representations Reflect Transition Statistics When the agent’s transition statistics mimic different behaviors, TEM learns new representations (left to right: different cells; top to bottom: environments 1, 2, real data). (A) When biased to move toward objects (white dots) TEM learns structural cells with a vector relationship to the objects—object vector cells (Høydal et al., 2019). These cells generalize to all objects. (B) TEM hippocampal cells reflect this behavioral transition change with similar cells, though they do not generalize to all objects—landmark cells (Deshmukh and Knierim, 2013). (C) When biased toward boundaries, TEM learns border cell-like representations (Solstad et al., 2008).

Animals do not move by diffusion (Purcell, 1977). We next examined representations learned by TEM when trained with different behavioral paradigms (Figure 5, see STAR Methods for full experimental details). For non-diffusive transitions, we mimic animals that prefer to spend time near boundaries and approach objects. Here, because the transition statistics change, so do the optimal representations for predicting future location. Indeed, training TEM on these *behavioral* transition statistics leads to the emergence of new cellular representations; importantly, these are also found in rodents. Now medial entorhinal representations, g, in TEM include border cells (Solstad et al., 2008; Figure 5C) and cells that fire at the same distance and angle from any object (object vector cells (Høydal et al., 2019; Figure 5A) for the two cases, respectively. This is easily understood: in order to make next-state predictions, TEM learns predictive representations, with object vector cells predicting the next transition is toward the object—as is often behaviorally the case.

Critically, these TEM medial entorhinal cells also generalize, with TEM object vector cells generalizing to all objects both within and across environments. The cells do not represent objects themselves, but rather their predictions about transitions, and they do so in a way that generalizes, allowing immediate inferences in new environments. Notably, these same properties are observed in object vector cells in rodent MEC (Høydal et al., 2019; Figure 5A).

Similar cells exist in TEM’s hippocampal layer, p, with a crucial difference. Here, object-sensitive cells represent the vector to particular objects but do not generalize across objects (Figure 5B)—they represent the conjunction between the structural representation and the sensory data. These cells are reminiscent of “landmark” cells that have been recorded in rodent hippocampus (Deshmukh and Knierim, 2013).

Objects occur at random locations; thus, when representing the transition statistics of the environment, TEM’s medial entorhinal layer g arbitrarily *composes* object vector cell representations (at any location) along with grid and other medial entorhinal representations. These results suggest that the “zoo” of different cell types found in medial entorhinal cortex may be viewed under a unified framework, summarizing the common statistics of tasks into basis functions that can be flexibly *composed* depending on the particular structural constraints of the environment the animal/agent faces.

### Structural Knowledge Is Preserved over Apparently Random Hippocampal Remapping

**Figure 6:**
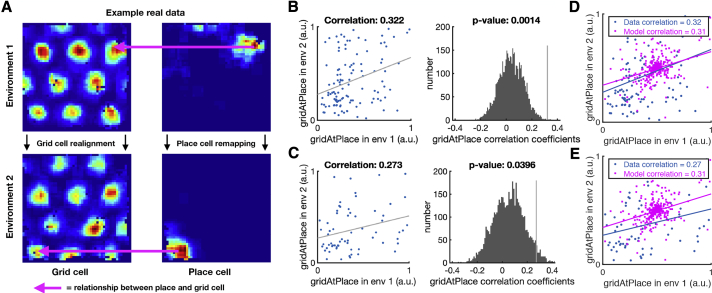
Structural Knowledge Is Preserved over Apparently Random Hippocampal Remapping (A) TEM predicts place cells remap to locations consistent with a grid code, i.e., a place cell co-active with a grid cell will be more likely to remap to locations where that grid cell is also active. (B and C) Data from open-field remapping experiments with simultaneously recorded place and grid cells (Barry et al., 2012; Chen et al., 2018). We compute the grid cell firing rate at the location of place cell peak for every grid cell, place cell pair in each of the two environments and then correlate this measure across environments (left). We compare this correlation coefficient to those computed equivalently but with randomly permuted place cell peaks (right). This is done for two independent datasets (B) (Barry et al., 2012) and (C) (Chen et al., 2018). The true observed correlation coefficients lies off the null distribution (p < 0.05), demonstrating place cell remapping is not random but instead tied to structural constraints of grid cells. (D and E) The same analysis on TEM learned representations shows qualitatively similar results to Barry et al. (2012) (D) and Chen et al. (2018) (E). See also Figures S6 and S7 and Tables S3 and S4.

The critical assumption that enables TEM’s structural inferences is that the hippocampal representations of new events are *not* random. Instead, they are constrained by learned structural representations in the entorhinal input. This assumption seems at odds with the commonly held belief that hippocampal place cells remap randomly between environments. However, the structural representation for space is *periodic*. Thus, place cells can preserve structural information across environments without being spatial neighbors with the same cells in each environment. Instead, individual cells need only to retain their *phases* with respect to the grid code. Here, structural knowledge is retained but remapping still occurs because place cells might, in a new environment, move to the same phase but with respect to a different grid peak (see e.g., Figure 6A). Together with the different sensory input between environments, this leads to remapping in TEM’s conjunctive hippocampal cells (Figure 4E).

Is this true in biological remapping? We tested data from two experiments in which both place and grid cells have been recorded while rats (Barry et al., 2012) and mice (Chen et al., 2018) freely forage in multiple environments. Experiment 1 (Barry et al., 2012) has two environments of the same dimensions (1 m by 1 m) but differing in their sensory cues so the animals could distinguish between them. Each of seven rats has recordings from both environments. Twenty-minute recordings are taken each day in both environments. Experiment 2 (Chen et al., 2018) has four mice in a real and a virtual reality environment of sizes (60 cm by 60 cm). Trials in the real and virtual environments were 20 and 40 min long, respectively.

**Figure S6:**
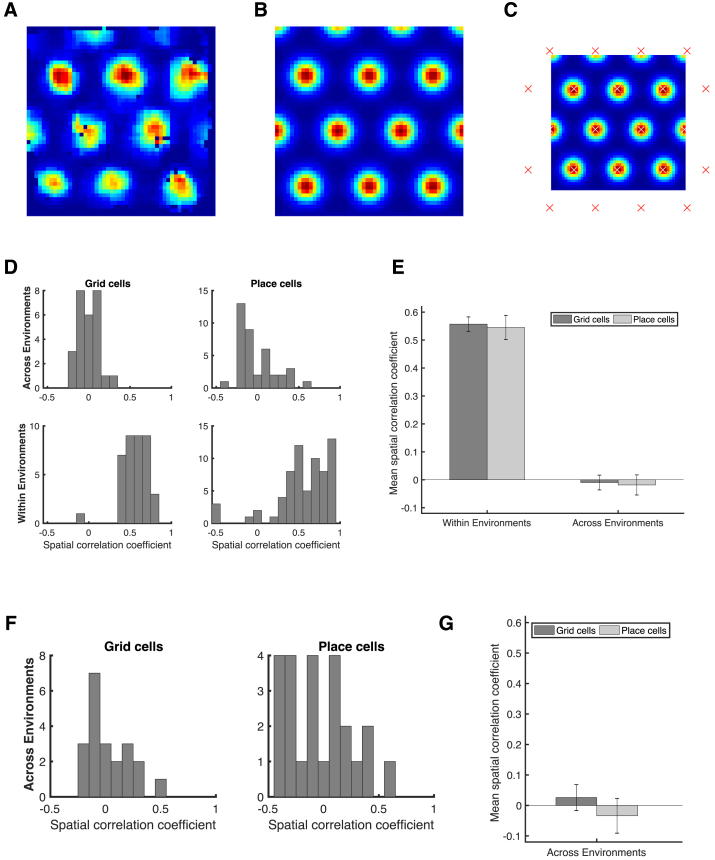
Fitting Ideal Grid Maps and Analysis of Real Data Showing Grid Cells Realign and Place Cells Remap, Related to Figure 6 **(A-C)** Ideal grid. We fit an idealized grid rate map using the formula from Stemmler et al. (2015) to the original grid cell rate maps to remove any possible confounds and to ensure that we obtain accurate grid cell peaks. (**A**) An example original grid cell rate map. (**B**) An idealized rate map fit to that in (**A**). (**C**) Accurate finding of grid cell peaks (white crosses) on the idealized grid rate map, which also allows peaks that extend outside the box to be used (red crosses). **D-E)** Grid realignment and place cell remapping across environments in dataset 1. (**D**) Histograms showing the distributions of spatial correlations for place and grid cells both within and across environments. (**E**) Bar plots showing the mean (± SEM) of these distributions. **F-G)** Grid realignment and place cell remapping across environments in dataset 2. (**F**) and (**G**) are same analyses as (**D**) and (**E**) but with dataset 2. They demonstrate distributions of spatial correlations near 0 for dataset 2. (**G**) has its axis locked to that of (**E**) for visualization.

**Figure S7:**
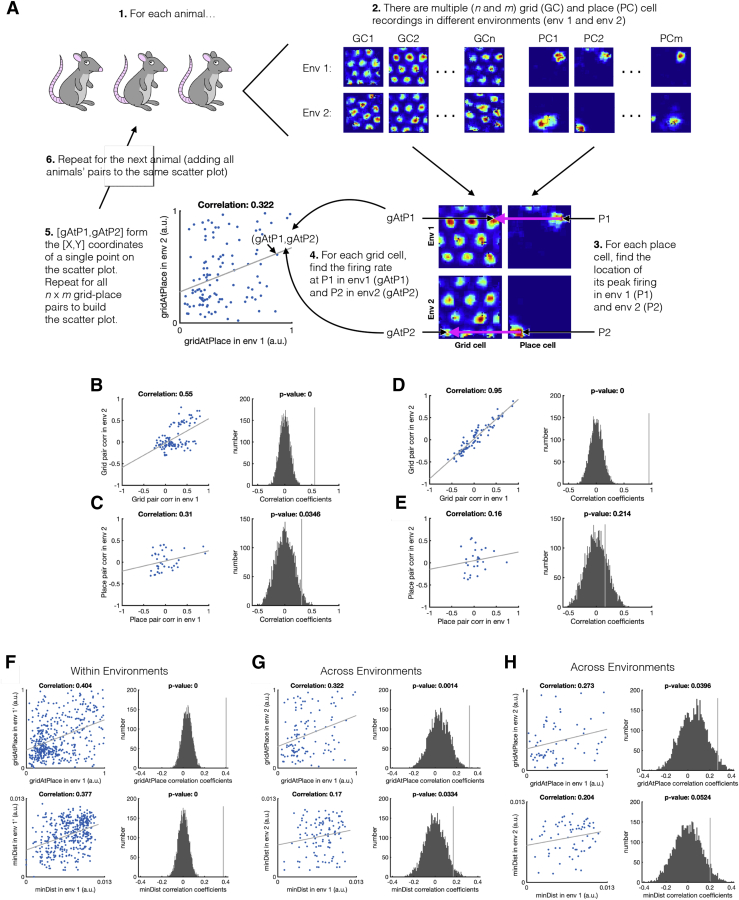
Schematic of Analysis Showing Preserved Grid-Place Relationships after Remapping, with Corresponding Results, Related to Figure 6 **A)** Schematic explaining the gridAtPlace analysis. Specifically how the scatterplot is generated. Note that in this figure original grid cell rate maps are shown, rather than ideal grid cell rate maps (Figures S6A–S6C) that were used to generate the main text figures. **B-C**) The grid cell correlation structure is preserved across environments in dataset 1. B) Dataset 1. Scatterplot shows the correlation across environments of the spatial correlations of grid cell-grid cell pairs (i.e., the correlation of the upper triangle of two grid cell by grid cell correlation matrices: one from environment 1 and one from environment 2). The histogram shows this correlation coefficient was significant relative to a null distribution of correlation coefficients obtained by permuting grid cell-grid cell pairs. (**C**) Same as A for place cells. **D-E**) Replication of preserved grid cell correlation structure across environments in dataset 2. D and E are the same format as (**B**) and (**C**). **F-G**) Preserved relationship between place and grid cells across environments in dataset 1. The scatterplots show the correlation of a given measure across trials, where each point is a place cell-grid cell pair. The histogram plots show where this correlation (gray line) lies relative to the null distribution of correlation coefficients. The p value is the proportion of the null distribution that is greater than the unshuffled correlation. (**F**) gridAtPlace (top) and minDist (bottom) measures are strongly significantly correlated over two trials within the same environment, as expected given the same place and grid code should be present. (**G**) These measures are also significantly correlated across the two different environments, providing evidence that place and grid cells retain their relationship across environments. (**H**) Replication of the preserved relationship between place and grid cells across environments in dataset 2. The gridAtPlace measure is significantly correlated at p<0.05 across real and virtual worlds and the minDist measure is trending very close to significance, replicating the preserved relationship between grid and place cells across environments.
2) **Does a given grid cell peak at a similar distance from the respective peaks of a given place cell in both environments?** For this “MinDist” measure, we do the same process as above, but X is now the minimum distance of a grid peak in env1 from P1, and Y is the minimum distance of a grid peak in env2 from P2. We normalize X,Y by grid scale of that grid cell. Note that the minDist measure is only calculated in analyses that fit idealized grids (to cells with grid score 0.8) to ensure that grid peaks are estimated effectively.

We asked whether the activity of each grid cell at the peak firing of each place cell peak (gridAtPlace) is correlated across environments (full details in the STAR Methods, Figures S6 and S7). If the place cell fires at the same grid phase in each environment, then the same grid cell will have strong activity at each place cell peak. To test the significance of this correlation, we perform a permutation test by generating a null distribution from randomly shifted place cells.

For experiment 1, we find significant correlation for the 115 within-animal place cell-grid cell pairs that satisfy conservative criteria (r=0.322, p < 0.01 [permutation test], Figure 6B) and for the liberal set of 255 pairs (r=0.63, p < 0.05). We replicate these results in dataset 2 across 64 conservative pairs (r=0.273, p < 0.05, Figure 6C) and the liberal set of 148 pairs (r=0.544, p < 0.05). These results are robust to all combinations of parameters settings for our criteria of cell acceptance to the analysis (Tables S3 and S4). We also show that an additional independent measure is significant for experiment 1 and trending for experiment 2 (STAR Methods).

If there were only a single grid frequency (or module) in entorhinal cortex, we would expect a near perfect correlation across environments between gridAtPlace scores for each grid-cell place-cell pair. While both datasets have non-zero correlations, the correlation is far from perfect (Figure 6). This would be expected if either (1) place cells are influenced by phases of more than a single grid module or (2) place cells predominantly received input from a single grid module, but we (the experimenter) do not know which module. Therefore, in order to gauge the magnitude of the effect, we performed the same analysis on TEM representations. Data and model show similar correlations (average $r_{data}=0.27-0.32$, $r_{model}=0.31$) (Figures 6D and 6E) .

These results demonstrate non-random place cell remapping in space and support a key prediction of our model: that hippocampal place cells, despite remapping across environments, retain their relationship with the entorhinal grid, providing a substrate for structural inference.

### A Mechanistic Understanding of Complex Non-spatial Abstractions

While cellular responses are well understood in rodent open-field tasks, we have little knowledge of how they combine to control behavior in more complex situations. Because TEM can learn arbitrary structural abstractions, it can also account formally for hippocampal and entorhinal responses in complex non-spatial tasks.

**Figure 7:**
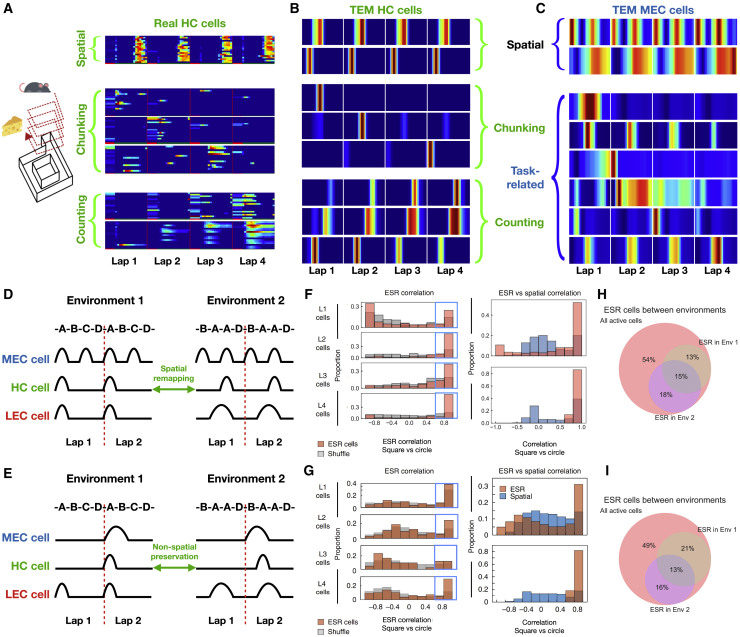
TEM Represents Non-spatial Reinforcement Learning Tasks and Predicts Non-spatial Remapping (A) In Sun et al. (2020), rodents perform laps of a track, only “rewarded” every 4 laps. Different hippocampal cell types are found: spatial place-like cells (top), those that preferentially fire on a given lap (middle), and those that count laps (bottom). (B) TEM learns similar representations when only “rewarded” every 4 laps. (C) TEM medial entorhinal cells learn both spatially periodic cells (top) and cells that represent the non-spatial task structure of “every 4 laps” (bottom). The latter cells are yet experimentally observed but are predicted by TEM. (D and E) TEM offers a mechanistic understanding of remapping in both spatial and non-spatial tasks. (D) Top/middle/bottom: schematic of entorhinal/hippocampal/sensory cells. Left/right: environment 1/2. Only two laps shown for clarity. TEM says spatial hippocampal cells are active when they receive input from both sensory (LEC; cell codes for A in this example) and MEC input. Place cells, thus, can only remap to other peaks (or within a broad MEC cell field) provided it also receives sensory input there. (E) TEM says, however, cells will retain their lap specificity despite spatially remapping (i.e., a lap 2 cell stays a lap 2 cell); since sensory observations repeat each lap, lap specificity is driven by MEC input. (F and G) Analysis to show TEM (F) and real (Sun et al., 2020) (G) hippocampal cells retain lap specificity after remapping. Left: distribution of lap-specificity correlations are significantly higher than shuffles. Top-right: distribution of spatial correlations (spatial) after remapping is compared to the distribution of lap-specific correlations (ESR). Bottom right: for cells of high lap-specific correlation after remapping (defined by the blue box in the left panel). (H) TEM prescribes which hippocampal cells will be active in each environment. The proportion of active cells that are ESR cells in environment 1, 2, or both (brown, purple, gray) imply approximate independence of cells recruited. (I) Data from Sun et al.(2020) showing the same effect^1^. Icons are from https://www.flaticon.com. See also Figure S5. .

To illustrate this, we consider a recent finding by Sun et al. (2020). Rodents perform laps of a circular track but only receive reward every four laps. Now hippocampal cells develop a new representation. While some cells represent location on the track, (i.e., place cells; Figure 7A, top), others are also spatially selective but fire only on one of the 4 laps (Figure 7A, middle). A third set fire again at a set spatial location but vary their firing continuously as a function of lap number (Figure 7A, bottom). Hippocampal cells maintain a complex combined representation of space and lap number.

When TEM was trained on this task, it learned these same 3 representations in the hippocampus (Figure 7B and S5G, see STAR Methods for further cells). Here “reward” was just a particular sensory event that repeated every 4 trials. As in the biological data, some TEM hippocampal cells encode location on every lap. These cells allow TEM to predict the sensory events that are unchanged between laps. However, as in recorded data, some TEM cells encode location on one of the four laps, and some with a lap gradient. These cells allow TEM to represent its position within the 4-lap cycle.

Importantly, TEM allows us to reveal a candidate mechanism. TEM’s medial entorhinal cells have reconfigured to code differently for each lap, understanding that the abstract task space is not a single lap but four (Figure 5C bottom). This mechanism is consistent with empirical data as manipulation of entorhinal cortex disrupts lap-sensitive hippocampal cells (Sun et al., 2020). However, TEM’s medial entorhinal representations stand as a prediction as Sun et al. (2020) did not record entorhinal cells.

These results suggest entorhinal cells can learn to represent tasks at multiple levels of cognitive abstraction simultaneously, with hippocampal cells reflecting their conjunction with sensory experience.

### Remapping in a Non-spatial Task

A machine that represents both space and non-space raises the possibility of studying remapping in non-spatial cells. Consider two examples of the 4-lap task with different sensory features. TEM says that, like their spatial counterparts, non-spatial hippocampal cells will remap to new *spatial* locations. However, unlike their spatial specificity, their non-spatial lap specificity will be preserved—they will preferentially fire on the same lap in each environment. To understand why, we remind ourselves of the mechanism behind TEM remapping for spatial cells (Figure 7D). Since a given hippocampal cell is only active when receiving both structural (MEC) and sensory (LEC) input, it can only move to new locations in the second environment where it also receives both MEC and LEC input. Exactly the same principle governs remapping in non-spatial cells (Figure 7E). Each cell remaps to a new spatial location, but since (1) its structural input is lap specific and (2) the sensory observations are the same for each lap, the cell will retain its lap specificity. In this case, TEM therefore predicts spatial remapping and non-spatial persistence via the same mechanism.

To quantify this, we analyze TEM representations from two sensorally different environments (details in STAR Methods). We quantify each cell’s lap preference using the ESR (event specific rate, following Sun et al.’s nomenclature of a lap as an event). This measure is a 4-vector containing the difference in activity between each lap and the average, at the peak location of the average lap. This vector therefore measures the between-lap (non-spatial) selectivity but ignores selectivity within lap (spatial). Again, following Sun et al. (2020), we measure the preservation of non-spatial activity in each cell by correlating this vector across environments (ESR correlation). By correlating the across-lap average activity, we can also measure the preservation of spatial representations.

Both in TEM and in Sun et al.’s mice, the distribution of ESR correlations (red) for hippocampal representations is concentrated toward high values for cells of all lap preference (Figures 7F and 7G, left) and is significantly different from shuffles (gray). Comparing ESR correlations to the more uniformly distributed spatial correlation shows hippocampal cells spatially remap but retain their lap specificity in both TEM and in mice (Figures 7F and 7G, top right), with this holding even when only using cells with high ESR correlations (Figure 7F, bottom right).

The same mechanism explains why spatial cells may be active in one environment but not another (Guzowski et al., 1999), as the relevant sensory and structural inputs may align in one environment but not the other. In both TEM and Sun et al. (2020)’s mice, this is also true for non-spatial cells (Figures 7H and 7I).

### Neural Predictions of the Tolman-Eichenbaum Machine

TEM further predicts a variety of yet unknown results. Here, we describe some key predictions and, where possible, point toward experiments that could validate such predictions.

#### LEC-Hippocampus Remapping

We have shown that structural inputs from the MEC are preserved across hippocampal maps during remapping (Figure 6). TEM also predicts that the relationship between the LEC and the hippocampus will be preserved across maps. Simultaneous recording from the LEC and the hippocampus in a remapping experiment should therefore reveal similar correlations to Figure 6.

#### Multiple Place Fields within an Environment

TEM predicts that place cells can have multiple place fields within an environment, as is observed experimentally (Rich et al., 2014). As this is due to the same mechanisms as remapping, TEM predicts that the correlations in the MEC firing observed between place fields in different environments in Figure 6 will also be observed for multiple place fields within environments. As above, this will also apply for the LEC.

#### Manipulation of Sensory Input by Virtual Reality

Consider a task where animals run 4 laps to get reward (as in Sun et al., 2020) but in virtual reality. If a section has a different appearance on each lap, interesting predictions can be made. In the variable section of the track, grid-cells, counting cells, and lap-specific cells will all exist in the MEC. In the hippocampus, however, cells will behave differently as MEC input will align with different sensory cells on each lap. All hippocampus cells will be lap specific in this section (no counting or place cells). This prediction is particularly stark and highlights TEM’s simplification that the hippocampus is solely responsible for binding. A softer prediction is that the lap specificity of all cells will increase in the hippocampus but not the MEC.

#### Task Representations and Latent States

As in the lapping mice, TEM predicts the properties of hippocampal cells will reflect a combined need to (1) predict the current sensory input and (2) separate states that predict different distant futures. They will thus contain representations of current location in space, but also current location in the task. Situations where sensory input is the same, but task location is different are referred to as latent states in reinforcement learning (Gershman and Niv, 2010). In general, TEM predicts latent-state-specific cells in any task. For example, existing representations of this type include splitter cells, inbound cells, and outbound cells in alternation tasks (Frank et al., 2000; Wood et al., 2000). TEM predicts that these cells will generalize across different sensory versions of the same task in the MEC but not in the hippocampus.

#### Structural Remapping

As shown, TEM predicts hippocampal remapping across two task environments with the same structure but different stimuli. However, TEM also says that the task’s sequential structure is implicitly encoded in the MEC code. Hence, it predicts MEC remapping (change in cell-cell correlation structure) when the task changes. This has been shown in specific situations (e.g., inclusion of walls [Derdikman et al., 2009; Gupta et al., 2014]), though TEM makes the general claim of remapping for changes in transition statistics. This may be most easily tested in primates where tasks need not be embedded in space. Recent fMRI evidence provides early support for this prediction (Baram et al., 2019). The correlation structure of spatial grid cells is thought to be highly stable (Yoon et al., 2013). Hence, in tasks that are embedded in space (as is typical in rodent tasks), while task representations may deform the spatial representation, they may instead be factored from (or built upon) the spatial representation. It will therefore be interesting to study task-remapping separately in both grid and non-grid MEC cells. While there is suggestive evidence that both of these mechanisms are possible in the context of changes in reward (Boccara et al., 2019; Butler et al., 2019), a formal test of this prediction would require a factorial design that independently varies sensory stimuli and task structure. Additionally, task statistics can be parametrically varied with TEM predicting structural remapping occurring gradually. For example, in sets of tasks where the animal spends different amounts of time near walls will affect the prevalence of border cell representations. It may also be interesting to study task remapping in prefrontal regions that provide input to the MEC (Baram et al., 2019; Hasselmo, 2005; Kaefer et al., 2020; Morrissey et al., 2017).

## Discussion

Building an understanding that spans from computation through cellular activity to behavior is a central goal of neuroscience. One field that promises such an understanding is spatial navigation and the hippocampus. However, while cells are precisely described for open-field foraging in small arenas, it has been unclear how these responses could generalize to real-world behaviors. Similarly, it has been unclear how to understand these spatial responses in the context of hippocampal involvement in memory broadly writ (Scoville and Milner, 1957) and relational memory in particular (Eichenbaum and Cohen, 2014). In this work, we have shown that by formalizing the problem of relational abstraction, using factorization and conjunction of representations, it is possible to account for spatial inferences as a special case of relational memory as hypothesized by Eichenbaum et al. (1999).

In doing so, we provided unifying principles that account for a number of seemingly disparate phenomena. For example, grid cells, band cells, border cells, and object vector cells all emerge from bases describing likely transitions. We show that this structural basis is also important for understanding several seemingly unrelated processes such as hippocampal remapping (Anderson and Jeffery, 2003; Bostock et al., 1991; Muller and Kubie, 1987; Lever et al., 2002) and transitive inference (Bunsey and Eichenbaum, 1996), which are shown to be two sides of the same coin—the structural knowledge transferred during remapping supports the generalization of transitive structure. While the idea that hippocampal memories are summarized in cortex is influential (McClelland et al., 1995), TEM therefore also suggests how cortical representations feed back onto the hippocampus to organize new experience and memory.

Recent related ideas have extended the predictive view of the hippocampus (Stachenfeld et al., 2017) to propose that entorhinal and frontal cortices provide bases for successor features at various hierarchical scales; more formally, entorhinal cortex is proposed to represent the eigenspace of successor features (Momennejad, 2020). This notion of abstracting predictive representations is shared with TEM. TEM demonstrates how generalization can happen using factorized and conjunctive representations, how this may relate to more traditional hippocampal views like path integration, and therefore how this process can be instantiated in the hippocampal-entorhinal loop. Further consideration of the relationships between these ideas may allow, for example, powerful generalizations in object feature—as well as relational—spaces.

It has been suggested that abstractions and structural learning may rely on hippocampal replay (Foster and Wilson, 2006; Lewis and Durrant, 2011; Liu et al., 2019) with sleep playing a crucial role in replay extracting regularities from wake experience. Notably, TEM uses a learning scheme similar to the wake sleep algorithm (Hinton et al., 1995) and Helmholtz machine (Dayan et al., 1995), which learn to sample from a generative model of the environment. The reliance on generative model predictions is notable as hippocampal replay appears to sample from a generative model of the environment (Evans and Burgess, 2019; Foster and Wilson, 2006; Stella et al., 2019; Vertes and Sahani 2019. Notably, TEM suggests that a fundamental role of replay and the hippocampal function more generally is the organization of sequences into structures (Buzsáki and Tingley, 2018; Schendan et al., 2003).

Though TEM makes predictions at the level of cells, its fundamental principles are at a computational level. However, by contrast to standard RNNs (Banino et al., 2018; Cueva and Wei, 2018), TEM takes anatomical considerations into account, allowing different predictions to be made for different cell populations and showing how they interact. Nevertheless, TEM is intended to provide insight and explanation at the computational level. In doing so, it ignores many known biophysical and anatomical properties of the hippocampal formation. It is not a biophysically realistic model.

TEM attempts to find general principles that include spatial reasoning. Notably, spatial reasoning provides a particularly clean example of the factorization of relational and sensory knowledge as well as a particularly powerful example of generalization, as relational meaning repeats regularly across space (O’Keefe and Nadel, 1978). Furthermore, the important role of spatial reasoning in evolution may provide particular pressures on representations that lead to efficient computations in space. However, by considering the relational memory problem more broadly, we have shown the same mechanism that can produce these spatial representations can also predict cellular responses in situations more complex than open-field foraging. While we have so far considered simple behaviors (e.g., running 4 laps of a maze to attain reward), we envisage that, together with exciting insights from reinforcement learning (Momennejad, 2020; Stachenfeld et al., 2017), this and similar frameworks may be useful in extending our computational understanding from open-field navigation toward Tolman’s original ideas of a systematic organization of knowledge spanning all domains of behavior (Tolman, 1948).

## STAR★Methods

### Key Resources Table

**Table:** 

REAGENT or RESOURCE	SOURCE	IDENTIFIER
**Deposited Data**		

Rodent data 1	Barry et al. (2012)	N/A
Rodent data 2	Chen et al. (2018)	N/A

**Software and Algorithms**		

MATLAB 2016b	MathWorks	https://www.mathworks.com/products/matlab.html
Python 3.6.1	Python Software Foundation	https://www.python.org/
Tensorflow 1.9.0	Abadi et al. (2016)	https://www.tensorflow.org/
Tolman-Eichenbaum Machine	This paper	https://github.com/djcrw/generalising-structural-knowledge

^1^ We note that Sun et al. (2020) determined active cells by those active on day 1 in environment 1, but from discussions with the authors these cells likely reflect the majority of all active cells

### Resource Availability

#### Lead Contact

Further information and requests for resources and reagents should be directed to and will be fulfilled by the Lead Contact, James Whittington (jcrwhittington@gmail.com).

#### Material Availability

This study did not generate new unique reagents.

#### Data and Code Availability

The model code generated during this study are available https://www.github.com/djcrw/generalising-structural-knowledge.

### Method Details

#### Task details

We wish to formalise a task type that not only relates to known hippocampal function, but also tests the learning and generalizing of abstract structural knowledge. This would then offer a single common framework (generalizing structural knowledge) that explains many hippocampal functions, and as it turns out, explains many known cell representations.

We formalise this via relational understanding on graph structures (a graph is a set of nodes that relate to each other). We demonstrate that two seemingly distinct hippocampal functions - spatial navigation and relational memory tasks - can be viewed in this common framework.

Should one passively move on a graph (e.g., Figure S1A), where each node is associated with a non-unique sensory observation (e.g., an image of a banana), then predicting the subsequent sensory observation tests whether you understand the graph structure you are in. For example, if you return to a previously visited node (Figure S1A pink) by a new direction - it is only possible to predict correctly if you know that a right→down→left→up means you’re back in the same place. Knowledge of such loop closures is equivalent to understanding the structure of the graph.

We thus train our model (TEM) on these graphs with it trying to predict the next sensory observation. TEM is trained on many environments sharing the same structure, e.g., 2D graphs (Figure S1A), however the stimulus distribution is different (each vertex is randomly assigned a stimulus). Should it be able to learn and generalize this structural knowledge, then it should be able to enter new environments (structurally similar but with different stimulus distributions) and perform feats of loop closure on first presentation.

The sensory stimuli are chosen randomly, with replacement, at each node. We understand that this is not like the real world, where adjacent locations have sensory correlations - most notable in space (though names in a family tree will be less correlated). Sensory correlations help with sensory predictions, thus if we use environments with sensory correlations, we would not know what was causing the learned representations, sensory correlations, or transition structure. To answer this question cleanly, and to know that transition structure is the sole cause, we do not use environments with sensory correlations.

We note that this feat of loop closure on first presentation has an intuitive meaning for space, but it is identical to the first presentation inferences made on tasks of transitive inference (Bunsey and Eichenbaum 1996) and social hierarchy (Kumaran et al., 2012) - tasks that the hippocampus plays a crucial role in.

In order to show that under a single framework (TEM), many aspects of hippocampus and entorhinal cortex can be explained, we thus choose graphs structures that reflect the types of tasks (space, transitive inference, social hierarchies) under which these brain areas have been studied. We now describe the details of each task. The details of simulations are in Simulation details.

#### Transitive inference

The hippocampus is crucial for problems of transitive inference with animals solving novel tasks on first presentation (Bunsey and Eichenbaum 1996). And so analogously we test whether TEM can learn about line structures and orderings i.e., if apple is one more than pear and pear is one more than monkey, what is 2 bigger than monkey?

To do so we use fully connected graphs, and order the nodes on a line i.e., label each node from 1 to K, where K is the number of nodes in the graph (Figure S1B). Each edge describes an action, e.g., the edge from 5 to 2 describes ‘below by 3′, the edge 4 to 14 describe ‘higher by 10’ etc. This structure and labeling of nodes and edges creates an implicit transitive hierarchy. We use lines of length {4, 5, 6} i.e., number of states {4, 5, 6}).

#### Social hierarchy

The hippocampus is known to be involved in reasoning over social hierarchies (Kumaran et al., 2012), and again we want to examine whether TEM is capable of learning the abstract set of relationships that govern social hierarchies.

We consider the graph of a family tree (Figure S1C). We limit ourselves to the case where each node has two children. We also eliminate the notion of gender - i.e., aunt/uncle is the same relationship, as is mother/father etc. Each edge corresponds to a family relationship i.e., ‘grandfather of…’. We use 10 types of relationships: {sibling, parent, grandparent, child 1, child 2, aunt/uncle, niece/nephew 1, niece/nephew 2, cousin 1 cousin 2}. We use {3, 4} levels of hierarchy i.e., number of states: {15, 31}.

#### 2D graphs

The hippocampus and entorhinal system has produced many famous cells, most notably those that have characteristic responses to space (Hafting et al., 2005; O’Keefe and Dostrovsky, 1971). Thus we consider graphs with spatial properties (e.g., Figure S1D). We consider both 4-connected and 6-connected graphs i.e., those with square or hexagonal symmetries. We use square environments of width 8-11 (number of states: {64, 81, 100, 121}), and hexagonal environments of edge width {5, 6, 7} (number of states: {61, 91, 127}).

#### Complex spatial tasks

Finally we consider non-spatial tasks embedded in a spatial world. We use the task set-up from Sun et al. (2020), where rodents perform laps of a circular track. Notably they are only rewarded every 4 laps. Thus the ‘true’ state space of the task is 4 laps not a single lap as space would suggest. This is a non-spatial task (every 4) embedded in a spatial world (circular track). We mimic this task on a loop graph of length l∗n, with *l* the lap length and *n* the number of laps (e.g., Figure S1E). The sensory observations are identical on each lap, however every *n* laps (i.e., every whole loop of the graph), the first state is a ‘reward’ state - where the reward is a unique sensory observation per environment. We use n=4 laps of length l=8.

#### The Tolman-Eichenbaum Machine

In the following description, we try to repeat the same information at successively increasing levels of detail. We hope this will allow readers to build an understanding of the model at their preferred level.

#### Problem statement - Intuitive

We are faced with the problem of predicting sensory observations that come from probabilistic transitions on a graph. The training data is a continuous stream of sensory observations and actions/relations (Figure S1A). For example, the network will see “banana, north, tree, east, book, south, door …” or “Joe, parent, Denise, niece, Anna, sibling, Fred ….” The model should predict the next sensory observation with high probability.

#### Problem statement - Formal

Given data of the form $D=\left\{\left(x_{\leq T}^{k},a_{\leq T}^{k}\right)\right\}$ with k∈{1,⋯,N} (which environment it is in), where $x_{\leq T}$ and $a_{\leq T}$ are a sequence of sensory observations and associated actions/relations (Figure S1A), *N* is the number of environments in the dataset, and *T* is the duration of time-steps in each environment, our model should maximize its probability of observing the sensory observations for each environment, $p_{\theta }\left(x_{\leq T}\right)$, where θ are the model parameters.

#### High level model description

We choose to model our problem probabilistically using a generative model - this allows us to offer a normative model for how the observed data depends on unobserved latent variables e.g., seeing a banana depends on where you are, but “where you are” is a latent variable - it is never observed. One can then in principle use Bayes’ theorem to invert this generative model and provide the optimal posterior distribution over the latent variables given the observations (“inference”). However, in most scenarios, including ours, this inversion is computationally impractical because the requisite integrals of the nonlinear functions cannot be solved analytically, and so **approximate** methods must be used (Bishop, 2006; MacKay 2003; Dayan and Abbott, 2001). Many such **approximate** methods have been proposed. One particularly powerful method is to learn the parameters of an “inference” model. Once trained, this model will approximately invert the generative model and perform the inference, mapping the observed data to the latent (unobserved) variables. This idea was introduced in the Wake-sleep algorithm (Hinton et al., 1995) and the Helmholtz machine (Dayan et al., 1995), and has since been adopted by Variational Autoencoders (Kingma and Welling, 2013; Rezende et al., 2014).

In common instantiations of generative models, latent variables are the “causes” of, for example, pixels in stationary images. Here, we provide a generative model where latent variables are positions that result from taking relational actions in a cognitive map. We further enable generalisation of knowledge across domains by separating latent variables of location that generalize across maps, g, from those that are ‘grounded’ in sensory experience and therefore specific to a particular map p. ‘grounded variables’, p encode [abstract location, sensory experience] conjunctions for the current environment.

The model aims to predict the next sensory experience from all previous sensory experiences. This problem is inherently *non-Markovian*. The next sensory experience can depend on historic experiences independently from the most recent experience (old locations might be encountered on meandering paths). However, the problem can be rendered *Markovian* by the inclusion of a Memory M that remembers what experience (x) is where (g) in the current environment. The inclusion of “grounded” variables, p, means that if good representations are learned at this level the memory simply has to remember them.

We give the full generative model for the general probabilistic case with noise in both the action and sensory inputs and derive the appropriate loss functions. However, in this manuscript we only consider tasks where there is no noise in these inputs. We therefore implement a version of the model that ignores noise (discussed in Optimization) - this leads to faster training and more accurate inference in the noise-free case.

#### TEM and the brain

We propose TEM’s abstract location representations (g) as medial entorhinal cells, TEM’s grounded variables (p) as hippocampal cells, and TEM’s sensory input x as lateral entorhinal cells. In other words, TEM’s sensory data (the experience of a state) comes from the ‘what stream’ via lateral entorhinal cortex, and TEM’s abstract location representations are the ‘where stream’ coming from medial entorhinal cortex. TEM’s (hippocampal) conjunctive memory links ‘what’ to ‘where’, such that when we revisit ‘where’ we remember ‘what’.

TEM’s medial entorhinal representation, (g), invites comparison to recurrent neural network models (RNNs) (Zhang, 1996), commonly used to model grid cell activity in spatial tasks (Fuhs and Touretzky, 2006; Guanella and Verschure, 2006; Burak and Fiete, 2009). Like these models, TEM’s g layer is a recurrent neural network and different recurrent weights mediate the effects of different actions/relations in changing the activity pattern in the network. Unlike these models, however, our weights are not hardcoded, but learnt from experience. Furthermore, due to the factorisation afforded by p, they can be learnt directly from sensory experience without any “location” input. They can therefore learn abstract map-like representations not only in spatial problems, but also in arbitrary non-spatial problems - even those in which it would be difficult for humans to hand code an effective “location” representation (such as a family tree).

TEM’s grounded variables (p) resemble hippocampal cells, encoding location-sensory conjunctions (Wood et al., 1999; Komorowski et al., 2009; Chen et al., 2019) and enabling fast episodic memories (Bostock et al., 1991; Wills et al., 2005; Nakazawa et al., 2002).

TEM’s sensory representations (x) resemble lateral entorhinal representations, encoding processed sensory input (here - objects) (Neunuebel et al., 2013; Deshmukh and Knierim, 2011; Manns and Eichenbaum, 2006). Notably, TEM learns most effectively if sensory representations are passed through an approximate Laplace Transform (Shankar and Howard, 2012) as is reported in lateral entorhinal cells (Tahvildari et al., 2007; Tsao et al., 2018) (see Inference architecture and Details about embedded hierarchy).

TEM describes the hippocampal-entorhinal system as one that performs inference; TEM medial entorhinal cells infer a location in abstract space based based on their previous belief of location (and optionally sensory information linked to previous locations via memory). TEM hippocampal cells infer the current memory representation based on a conjunction between the sensory data and believed location in abstract space.

Though we already refer to these variables as entorhinal and hippocampal cells, we reiterate that no representations are hard-coded - *all TEM representations are learned*.

#### High-level algorithmic description

We now describe the fundamentals behind the Tolman-Eichenbaum Machine (TEM). TEM sees a stream of sensory observations and actions (x and a). It’s objective is to predict the next sensory input. If these observations are arranged on a graph with any regularities, TEM can profit from these regularities to predict the sensory consequences of edges it has never previously taken. After learning these regularities, TEM can transfer them to new environments that have the same regularities, but different sensory observations.

#### Principles

TEM relies on two simple principles / components. First a map-like component that learns about the abstract structure shared across environments (Tolman), and second a conjunctive memory component that grounds this learned abstract structure to the current environment (Eichenbaum). We denote the map-like variables as g, and the grounded conjunctive variables as p.

Each grounded variable p is a conjunction, tying an abstract location g to a sensory experience x. Each abstract location, however, has the potential to instantiate many different grounded variables - one for each for possible sensory experience. An attractor network memory learns, after a single experience, which location-experience pair is valid in the current world. The opposite is also true - a sensory experience can re-instantiate the memory of a grounded variable i.e., the conjunctive memory process allows both abstract location to predict sensory experience, and sensory experience to predict abstract location.

Naturally, TEM can only predict a sensory observation should it have seen it before and formed a memory of its grounded variable. TEM re-instantiates memories of grounded variables via indexing from its abstract location, g, and so re-instantiating the correct grounded variable requires TEM to index using the same abstract location code as when the memory of grounded variable was formed.

This puts strong constraints on the types of representations TEM must learn. First, it must learn a structural map-like code that transferably path-integrates such that g is the same when returning to a state (so the correct memory is indexed). Second it must learn representations g that are different for different states - so that each state can have a separate memory attached to it. These two constraints are fundamental to TEM representations, and are shown to be satisfied by grid-cell and other entorhinal codes.

#### Generative model

The generative model sees an action a, combines this with its previous g to predict the next abstract location in its cognitive map g which then proposes candidate grounded variables. An attractor network pattern completes these candidates, suppressing those that have not been experienced before, to restore a memory of the appropriate grounded variable p. The restored memory/grounded variable then predicts sensory observation x. This is presented as a graphical model (Figure S2A) and also schematically (Figure S3).

#### Inference Model

The inference model sees a sensory observation x, retrieves a memory of grounded variable best related to this sensory observation, then infers the next g from both the previous g (and action a) and this memory of grounded variable. p is then re-inferred using the best estimate of g and the new x. This new grounded variable p is used to update memory weights, *M*. This is presented as an inference model (Figure S2B) and also schematically (Figure S4).

#### Training

Both the generative and inference models have weights that must be learnt. The objective of training is for the generative model to predict the sensory input, x, and for the inference model to infer the generative model’s latent variables, [p, g], from the sensory input. The resulting training algorithm (Figure S2C) involves an interplay between generative and inference models, in which the generative model takes the current state of the inference model and (from this) predicts its next state (including the next sensory data). This leads to errors between the predicted and inferred/observed variables at each level [p, g, x]. The weights in both networks are adjusted along a gradient that reduces these errors using backpropagation through time.

The model is trained in multiple different environments, differing in size and sensory experience. When entering a new environment, network weights are retained but Hebbian weights M are reset. The most important weights are those that transition g as they encode the structure of the map. They must ensure (1) that each location in the map has a different g representation (so a unique memory can be built), (2) that arriving at the same location after different actions causes the same g representation (so the same memory can be retrieved) - a form of path integration for arbitrary graph structures. For example, the relation “uncle” must cause the same change in g as father followed by brother, but different from brother followed by father (‘non-associative’ relationships, not just associative ones seen in 2d graphs). These transition weights are shared between generative and inference models, though other weights are not. Shared weights are atypical for variational autoencoders, but are important for biological considerations. At each step we compared what was inferred to what was predicted from the inferred at the previous time-step - we add up the losses for a sequence and then update the weights.

#### Hierarchies in the map

When representing tasks that have self repeating structure (as ours do), it becomes efficient to hierarchically organize your cognitive map. In this spirit, we separate our model into multiple parallel streams, each as described above (i.e., each stream takes in x and temporally smooths it (each stream with a different learned smoothing constant), each stream’s g can transition via path integration and each stream’s p is a conjunction between the g and the filtered x), where each stream can learn weights to represent the world at different scales. These streams are only combined when retrieving memories (grounded variables p) in the attractor network. We provide further details on this in Details about embedded hierarchy.

#### Model flow summary

The inference model is the one that sees sensory data $x_{t}$ at each time-step *t*. It is ‘awake’ and transitions through time on its own inferring $g_{t}$ and $p_{t}$ at each time-step. The inference model infers the new abstract location $g_{t}$ before inferring the new grounded variable $p_{t}$. In other words latent variables g and p are inferred in the following order $g_{t}$, $p_{t}$, $g_{t+1}$, $p_{t+1}$, $g_{t+2}\cdots$. This flow of information is shown in a schematic in Figure S2 green.

Independently, at each time-step, the generative model asks ‘are the inferred variables from the inference model what I would have predicted given my current understanding of the world (weights)’. I.e., 1) Is the inferred $g_{t}$ the one I would have predicted from $g_{t-1}$. 2) Is the inferred $p_{t}$ the one I would have predicted from $g_{t}$. 3) Is $x_{t}$ what I would have predicted from $p_{t}$. This leads to errors (at each timestep) between inferred and generative variables $g_{t}$ and $p_{t}$, and between sensory data $x_{t}$ and its prediction from the generative model.

At then end of a sequence, these errors are accumulated, with both inference and generative models updating their parameters along the gradient that matches each others variables and also matches the data.

Since the inference model runs along uninterrupted, it’s activity at one time-step influence those at later time-steps. Thus when learning (using back-propagation through time - BPTT), gradient information flows backward in time. This is important as, should a bad memory be formed at one-time step, it will have consequences for later predictions - thus BPTT allows us to learn how to form memories and latent representations such that they will be useful many steps into the future.

#### Detailed algorithmic description

##### Generative architecture

TEM has a generative model (Figure S2A) which factorises as

$$p_{\theta }\left(x_{\leq T},p_{\leq T},g_{\leq T}\right)=\prod_{t=1}^{T}p_{\theta }\left(x_{t}|p_{t}\right)p_{\theta }\left(p_{t}|M_{t-1},g_{t}\right)p_{\theta }\left(g_{t}|g_{t-1},a_{t}\right)$$

$M_{t-1}$ represents the agent’s memory composed from past hippocampal representations $p_{<t}$ . θ are parameters of the generative model. The initial $p_{\theta }\left(g_{1}|g_{0},a_{1}\right)=p_{\theta }\left(g_{1}\right)$, i.e not conditioned on any prior variables. The model can be described by a sequence of computations represented by the following equations:

**Table:** 

State transition	$g_{t}∼N\left(\cdot |\mu =f_{g}\left(g_{t-1}+W_{a}g_{t-1}\right),\sigma =f_{\sigma_{g}}\left(g_{t-1}\right)\right)$
Entorhinal input to hippocampus	${\tilde{g}}_{t}=W_{repeat}f_{down}\left(g_{t}\right)$
Retrieve memory	$p_{t}∼N\left(\mu =attractor\left(g_{t},M_{t-1}\right),\sigma =f\left(\mu \right)\right)$
Sensory prediction	$x_{t}∼Cat\left(f_{x}\left(p_{t}\right)\right)$
Repeat process for the next timestep	$\to g_{t+1}\to p_{t+1}\to x_{t+1}\cdots$

We pictorially show these processes in Figure S3 (just consider the blue stream initially, the second red stream will make sense in Details about embedded hierarchy). We note that the various weights used in the network are describes in Table S2.

To predict where we will be, we can transition from our current location based on our heading direction (i.e., path integration). $W_{a}$ is a set of learnable weights for each available action (or alternatively the output of an MLP with a as its input) and $f_{g}$ is a activation functions that thresholds at ± 1.

Once TEM has transitioned, it then retrieves a memory indexed by it’s believed location. Memories are retrieved via an attractor network (details Retrieval using an attractor network). $f_{\sigma_{g}}$ is a simple multi layer perceptron (MLP).

After the memory has been retrieved, sensory information is extracted in order to predict the current observation. Our sensory sensory data is represented in a one-hot encoding (a vector with a single entry of 1 and all other entries 0) where each element in the vector corresponds to a different sensory experience, and so we model it with a categorical distribution Cat. The function $f_{x}\left(\cdots \right)$ is $softmax\left(f_{d}\left(w_{x}W_{tile}^{T}p_{t}+b_{x}\right)\right)$, where $W_{tile}$ is a fixed matrix (described in Inference architecture), $w_{x}$ is a scalar weight, and $f_{d}$ is a MLP for ‘decompressing’ into the correct input dimensions.

#### Inference architecture

We have just defined the generative model, however to do anything interesting we need to be able to infer the posterior over the hidden variables. Unfortunately, due to the inclusion of memories, as well as other non-linearities, the posterior $p_{\theta }\left(g_{t},p_{t}|x_{\leq t},a_{\leq t}\right)$ is intractable. We therefore turn to approximate inference, and in particular the variational autoencoder framework (Kingma and Welling, 2013; Rezende et al., 2014). Here the inference distribution is parametrised by a neural network, which during training learns how to infer.

The split between inference and generative networks is analogous to the idea of the sleep-wake algorithm. The inference network is ‘awake’ and observes the world, seeing each state as it transitions through the environment. The generative network is used during ‘sleep’ for *training* and where it compares ‘sleep’ generated variables to the inferred ‘awake’ ones. This allows training of *both* networks such that the inference network and generative network learn to align themselves i.e., the generative network learns to predict both sensory data and the variables inferred by the learned inference network (a.k.a recognition distribution) which, in turn, learns to appropriately map sensory events to latent variables.

In defining our approximate recognition distributions, $q_{\varphi }\left(\cdots \right)$, we make critical decisions that respect our proposal of map-structure information separated from sensory information as well as respecting certain biological considerations. We use a recognition distribution that factorises as

$$q_{\varphi }\left(g_{\leq T},p_{\leq T}|x_{\leq T}\right)=\prod_{t=1}^{T}q_{\varphi }\left(g_{t}|x_{\leq t},M_{t-1},g_{t-1},a_{t}\right)q_{\varphi }\left(p_{t}|x_{\leq t},g_{t}\right)$$

See Figure S4 for inference model schematic. *φ* denote parameters of the inference network. The variational posterior can be expressed by the following equations.

**Table:** 

Compress sensory observation	$x_{t}^{c}=f_{c}\left(x_{t}\right)$
Temporally filter sensorium	$x_{t}^{f}=\left(1-\alpha^{f}\right)x_{t-1}^{f}+\alpha^{f}x_{t}^{c}$
Sensory input to hippocampus	${\tilde{x}}_{t}=W_{tile}w_{p}f_{n}\left(x_{t}^{f}\right)$
Retrieve memory	$p_{t}^{x}=attractor\left({\tilde{x}}_{t},M_{t-1}\right)$
Infer entorhinal^2^	$g_{t}∼q_{\varphi }\left(g_{t}|p_{t}^{x},g_{t-1},a_{t}\right)$
Entorhinal input to hippocampus	${\tilde{g}}_{t}=W_{repeat}f_{down}\left(g_{t}\right)$
Infer hippocampus	$p_{t}∼N\left(\cdot |\mu =f_{p}\left({\tilde{g}}_{t}\cdot {\tilde{x}}_{t}\right),\sigma =f\left({\tilde{x}}_{t},{\tilde{g}}_{t}\right)\right)$
Form memory	$M_{t}=hebbian\left(M_{t-1},p_{t}\right)$
Repeat process for next observation	$\to x_{t+1}\to g_{t+1}\to p_{t+1}\cdots$

We pictorially show this process (with no Hebbian memory storage) in Figure S4 (just consider the blue stream initially, the second red stream will make sense in Details about embedded hierarchy). We now explain step by step in words, offering further details and hopefully some intuition.

We take our input $x_{t}$, which is a one-hot encoding of sensory experience (e.g., each element in the vector corresponds to a different sensory experience), and compress it via $f_{c}\left(x_{t}\right)$. We compress from a one-hot to a two-hot encoding to reduce the size of the resulting network and ease computation (shown in Figure S4).

We then smooth this compressed representation over time using exponential filtering with filtering parameter $\alpha^{f}$. We note that although the exponential smoothing appears over-simplified, it approximates the Laplace transform with real coefficients. Cells of this nature have been discovered in LEC (Tahvildari et al., 2007; Tsao et al., 2018).

Next, we normalize the representation using $f_{n}\left(x_{t}^{f}\right)$ which demeans then applies a rectified linear activation followed by unit normalization. These representations are then scaled by the scalar weight $w_{p}$, and then multiplied by a fixed weight matrix $W_{tile}$ (which gives the appropriate hippocampal dimension - all dimensions shown in Table S2) to give ${\tilde{x}}_{t}$ which is TEM’s sensory input to hippocampus.

We are now ready to infer where we are in the graph. We factorise our posterior on g as

$$q_{\varphi }\left(g_{t}|x_{\leq t},M_{t-1},g_{t-1},a_{t}\right)=q_{\varphi }\left(g_{t}|g_{t-1},a_{t}\right)q_{\varphi }\left(g_{t}|x_{\leq t},M_{t-1}\right)$$

To know where we are, we can path integrate (the first distribution, equivalent to the generative distribution described above) as well as use sensory information that we may have seen previously (second distribution). The second distribution (optional) provides information on location given the sensorium. Since memories link location and sensorium, successfully retrieving a memory given sensory input allows us to refine our location estimate. We use ${\tilde{x}}_{t}$ as the input to the attractor network to retrieve the memory associated with the current sensorium, $p_{t}^{x}$. We use MLPs with input $W_{repeat}^{T}p_{t}^{x}$ to parametrise the mean and variance of the distribution ($W_{repeat}$ is a fixed matrix described below). This factored distribution is a Gaussian with a precision weighted mean - i.e., we refine our generated location estimate with sensory information.

For Bayesian connoisseurs, we note that, unlike $p_{t}$, these retrieved memories $p_{t}^{x}$ are not random variables in the generative model and are therefore not inferred. Instead they are part of the function in the inference model that learns the approximate posterior on $g_{t}$. Nevertheless they share similarities to $p_{t}$, e.g., they have the same dimension and are pressured to learn similar representations (see Section ‘Optimization’). Biologically, they can be thought of as memories cued only by sensory input, and not inferred from the combination of sensory and structural input.

Now that we have inferred where we are, we are ready to form a new memory - infer our hippocampal representation. After the the entorhinal representation is down-sampled using $f_{down}\left(\cdots \right)$, we then multiply by a fixed weight matrix $W_{repeat}$ (which gives the appropriate hippocampal dimension - all dimensions shown in Table S2) to give ${\tilde{g}}_{t}$. We define the mean of the inferred hippocampal representation as the element wise multiplication of ${\tilde{x}}_{t}$ and ${\tilde{g}}_{t}$ followed by an activation function. We choose the leaky rectified linear unit activation function (additionally threshold at ±1) to create sparsity and ensure the only active hippocampal cells are those that receive both map-structure and sensory information. We note that the two fixed weight matrices are designed such that their application, followed by an element wise product between ${\tilde{x}}_{t}$ and ${\tilde{g}}_{t}$, is equivalent to an outer product followed by reshaping to a vector (Figure S4 bottom-left).

#### Memories

##### Storage using Hebbian learning

Memories of hippocampal cell representations are stored in Hebbian weights between hippocampal cells. We choose Hebbian learning, not only for its biological plausibility, but to also allow rapid learning when entering a new environment. We use the following learning rule to update the memory:

$$M_{t}=\lambda M_{t-1}+\eta \left(p_{t}-{\hat{p}}_{t}\right){\left(p_{t}+{\hat{p}}_{t}\right)}^{T}$$

where ${\hat{p}}_{t}$ represents place cells generated from inferred grid cells. λ and η are the rate of forgetting and remembering respectively. We note than many other types of Hebbian rules also work.

Notably, unlike the generative network, there is no requirement for a memory in the inference network. However, including such a memory allows the network to refine the path integration with landmark information *before* creating its place code and therefore speeds learning dramatically. However, representations in the main paper are observed both in networks that include an inference memory and those that do not.

In networks that do use an inference memory, we can either use the same memory matrix as the generative case (as the brain presumably does), or we can use a separate memory matrix. Best results (and those presented) were when two separate matrices were used. We used the following learning rule for the inference based matrix: $M_{t}^{x}=\lambda M_{t-1}^{x}+\eta \left(p_{t}-p_{t}^{x}\right){\left(p_{t}+p_{t}^{x}\right)}^{T}$, where $p_{t}^{x}$ is the retrieved memory with the sensorium as input to the attractor.

##### Retrieval using an attractor network

To retrieve memories, similarly to Ba et al. (2016), we use an attractor network of the form

$$h_{\tau }=f_{p}\left(\kappa h_{\tau -1}+M_{t-1}h_{\tau -1}\right)$$

where τ is the iteration of the attractor network and κ is a decay term. The input to the attractor, $h_{0}$, is from the grid cells or sensorium (${\tilde{g}}_{t}$ or ${\tilde{x}}_{t}$) depending on whether memories are being retrieved for generative or inference purposes respectively. The output of the attractor is the retrieved memory. We choose the number of iterations as 5.

#### Details about embedded hierarchy

Though not a requirement, we embed TEM with the notion of hierarchical scales. TEM abstract location and grounded variable (memory) representations, $g_{t}$ and $p_{t}$ respectively, now come in different streams (hierarchies/modules) indexed by superscript *f* - $p_{t}^{f}$ and $g_{t}^{f}$. This allows the learning of higher frequency statistics of the environment that can be reused across learned lower frequency statistics, improving the speed of learning and reducing the number of weights that need to be learnt. Additionally, the separation into hierarchical scales helps to provide a unique code for each position; even if the same stimulus appears in several locations of one environment, since the surrounding stimuli, and therefore the larger scale hippocampal cells, are likely to be different.

TEMs hierarchy is consistent with the hierarchical scales observed across both grid cells (Stensola et al., 2012) and place cells (Kjelstrup et al., 2008), and with lateral entorhinal cortex receiving sensory information in hierarchical temporal scales (Tsao et al., 2018).

Implementation wise, this means our network has several parallel streams of the procedure described above, each indexed by the superscript *f*. Each stream has its own learnable parameters (e.g., temporal filtering coefficients in the approximate Laplace transform $\alpha^{f}$ - a smaller $\alpha^{f}$ means a longer temporal smoothing window). Each stream also uses its own $W_{tile}^{f}$ and $w_{p}^{f}$. We schematically show an example of two separate streams in Figures S3 and S4for the generative and inference network respectively.

We use 5 parallel streams, indexed stream 1-5. Each stream receives the same input and is identical except for the following points.

1 The input to each stream is smoothed with an exponential kernel that is learnt from the data. Each stream can therefore learn a different smoothing kernel (leading to different temporal scales). (Note that a population of exponential kernels forms a Laplace Transform with real coefficients, as observed in LEC in rodents [Tahvildari et al., 2007; Tsao et al., 2018] and monkeys [Bright et al., 2020]).
2 We build in an asymmetry in the connectivity of the memory attractor, so that memory recollection is in a particular order (see below). This sets up a situation where the network can profit from learning different *temporal scales* when smoothing x for each stream, and different *spatial frequencies* for each stream’s structural representation g. Memories are most stable if large scales can influence small scales but not vice versa (the memory first attracts to the gist and then fills in the details). This does not affect accuracy but improves learning speed.
3 The number of neurons are smaller in the streams with higher index (large scales need fewest place cells).
4 The place cells from stream 1 predict the raw unsmoothed sensory data (see Figure S3). Stream 1 is then encouraged to learn small scales, as it must change rapidly to predict different observations at adjacent nodes.

Hence, although all of the scales in the model are learnt (and could in principle all be the same), these conditions ensure that the model profits from learning a hierarchy.

The asymmetry in the memory retrieval is as follows (asymmetry only for the generative memory)

1 M is only allowed connections from stream $f^{\prime }$ to *f*, where $f^{\prime }\geq f$, i.e., M is an upper triangular block matrix. This means that stream 1 receives information from all other streams, whereas stream 5 only receives information from steam 5. This biases smaller scale information in the streams with lower *f* indices (e.g., streams 1-2), and larger scale information in the streams with higher *f* index (e.g., streams 3-5)
2 The attractor, in the generative network, stops early for the streams with higher index. Stream 5 only gets 1 iteration, stream 4 gets 2 iterations, and stream 1 gets 5 iterations.

One can also use an asymmetry in the state transitions. By default, each stream has its own $W_{a}$ (the action dependent transitions weight from $g_{t-1}$ to $g_{t}$), though a similar hierarchy to the connections in M can be imposed. These asymmetries do not affect accuracy but do improves learning speed.

#### Weights in the network

For clarity, we discuss the different weights used in the network. First though, there is an important distinction in the types of weights spoken about; network weights and Hebbian weights. Network weights are learned by backpropagation, whereas Hebbian weights updates are implemented in the forward computations of the network itself - this is why these weights can store memories (Ba et al., 2016). These forward computations get ‘back-propagated’ through and provide the network weights with appropriate error signals - thus backprop (gradient descent) must learn how to use Hebbian learning.

The different explicitly mentioned weights in the network are detailed in Table S1. We note that there will be other weights matrices in TEM where we have used a MLP function, though they simply serve as function approximators.

#### Interplay of backprop and Hebbian learning

What does it mean that ‘backprop (gradient descent) must learn how to use Hebbian learning’?

##### Intuitive answer

The “abstract location” cells and sensory representations are the input to the Hebbian Matrix that form the memory. At recall time, to recall the right sensory code, you need to activate the right ‘abstract location’ cell representation. If you have never taken this path to that location, you need to path integrate to activate the right cell representation. This is what we mean by “Learning to use the memory.” It needs to form representations which will put the right activity pattern on the input of the memory at the right time. This is difficult because we want to learn them via gradient descent, but the gradients depend on what is in the memory! It is solved using backpropagation through time where the partial derivatives **account for the effect of the memory**. This means the memory itself must be differentiable, even though we are not optimizing its Hebbian weights. This is what we meant by ‘thus backprop (gradient descent) must learn how to use Hebbian learning’

##### Machine learning answer

The trick is that the computations of Hebbian updates (as well as the attractor dynamics) can be embedded in the forward propagation of an ANN. So the whole process of taking sensory and action inputs at each time-step, inferring the grid cells, inferring the place cells, path integration, Hebbian memories and retrieving memories is just one BIG artificial neural network. All of those aforementioned computations are differentiable, and so, on receiving a training signal, backprop can pass gradients though the whole big ANN and update the **network** weights. Backprop learning the **network** weights can then be seen as a slow ‘outer loop’ learning of general structure, whereas the **Hebbian** learning is the fast ‘inner loop’ learning the particular world.

#### Summary of model key points

1 The environments are graph structures, and the ‘agent’ wanders around from node to node observing a single sensory object at a time at each node. The agent must try to predict the sensory experiences at each time step.
2 This is not a reinforcement learning problem as the agent does not choose the actions. They are provided. Instead it is a sensory prediction problem.
3 The model is not given any location information. The only information it is given is the current sensory input and the actions/relation that leads to the next sensory input.
4 Sensory input does not contain any information about spatial location. The sensory input is simply a (1-hot) vector of length N (number of objects) with a single non-zero element identifying which object is currently being experienced. This 1-hot vector is compressed to make a distributed representation at the input of LEC, and then subsequently temporally smoothed. There is no information about location in the input.
5 Although we use graphical models to describe the **problem**, the network itself is not a graph neural network. It has no notion of edges or nodes, as in GNNs. It simply predicts the next observation in time. The network consists of two parts - 1) a path integration network that must learn to predict the next memory 2) a memory network that combines the location of the path integrator with the current sensory stimuli to form a memory of what was observed where.
6 All representations are learnt. We called g abstract locations as they learn knowledge only about the structure of the problem. We called p grounded locations, not because they are supplied to the model, but because, unlike abstract locations, they are “sensorially grounded.” That is, the network ties them to particular sensory experiences. We have clarified terminology in answers below, but it is critical to understand that all representations are learnt.
7 Actions are provided in the form of a one-hot vector and the network must learn to understand these vectors in a meaningful way. For example it must learn that transitioning a g representation first by a ‘right’ action and second by a ‘left’ action produces the same g representation - this is not an obvious realization from a one-hot vector alone. Similarly in family trees, a one-hot vector provides no information as to how the relations add up to mean the same thing e.g., mother + father = uncle. This is the true understanding of a family tree and that is what the network has to learn.
  (i) To see this, consider the following: there are 10 different relations in our family tree. These are supplied to the network in 10-vectors. So the relation grandparent might be [0 0 1 0 0 0 0 0 0 0]. There are also 45 sensory elements [people’s names], so Jim might be [0 0 0 0 0 0 1 0 0 0 …0 0] (45 elements). At each timestep, these two vectors are the only things we give to the network. It has to learn everything else. Because Jim may be a parent and a grandparent and a brother and an uncle, different series of relations from different starting points can lead to Jim. To predict when the network will see Jim, it needs to understand how these different relations relate to each other. This structural knowledge is implicitly represented in the recurrent weights between g, such that the units g act as a location in an abstract family tree. But g knows nothing about who is at that location. To recall who is at that location g must index p. p binds together the abstract location with the name “Jim.” We call it grounded because it is grounded in sensory experience.
  (ii) This mimics the classic distinction between cortical (network) and hippocampal (Hebbian) learning expressed, for example, in complementary learning systems (McClelland et al., 1995) or by Marr, but here the network weights are optimized to control the Hebbian weights and therefore build efficient memories.
8 There are two types of weight. *Network* weights are learned through error backpropagation and are updated after one or many runs through an environment. *Hebbian* weights are adjusted as part of the network computation on each timestep. They are how the network remembers what is where in a new map. The *network* weights must learn to generalize relational structure across environments. They act like weights in a modern recurrent neural network and are fixed when the network is run. The *Hebbian* weights are never fixed. They constantly change to store the most recent memories of the network within an environment.

#### Optimization

We wish to learn the parameters for both the generative model and inference network, θ and φ, by maximizing the evidence lower bound (ELBO), a lower bound on $\text{ln}p_{\theta }\left(x_{\leq T}|a_{\leq T}\right)$. Following Gemici et al. (2017) (see Whittington et al., 2018 supplementary material), we obtain a free energy

$$F=\sum_{t=1}^{T}E_{q_{\varphi }\left(g_{<t},p_{<t}|x_{<t}\right)}\left[J_{t}\right]$$

where

$$J_{t}=E_{q_{\varphi }\left(\ldots \right)}\left[\ln\;p_{\theta }\left(x_{t}|p_{t}\right)+\text{ln}\frac{p_{\theta }\left(p_{t}|M_{t-1},g_{t}\right)}{q_{\varphi }\left(p_{t}|x_{\leq t},g_{t}\right)}+\text{ln}\frac{p_{\theta }\left(g_{t}|g_{t-1},a_{t}\right)}{q_{\varphi }\left(g_{t}|x_{\leq t},M_{t-1},g_{t-1},a_{t}\right)}\right]$$

as a *per time-step* free energy. We use the variational autoencoder framework (Kingma and Welling, 2013; Rezende et al., 2014) to optimize this generative temporal model. The three error terms of this equation are depicted as orange arrows in Figure S2.

Up to this point the model definition is probabilistic and capable of a Bayesian treatment of uncertainty. However, in the tasks examined in this manuscript there is no uncertainty, so there is no need for this complexity. Hence, we use a network of identical architecture but only using the means of the above distributions - i.e., not sampling from the distributions. We then use the following surrogate loss function to mirror the ELBO:

$$L_{total}=\sum_{t=1}^{T}L_{x_{t}}+L_{p_{t}}+L_{g_{t}}$$

with $L_{x_{t}}$ being a cross entropy loss, and $L_{p_{t}}$ and $L_{g_{t}}$ are squared error losses between ‘inferred’ and ‘generated’ variables - in an equivalent way to the Bayesian energy function.

It is also possible to speed up learning with some augmented losses: As part of $L_{x_{t}}$ we include losses between sensory experience and 3 different generative model predictions - those generated directly from the inferred $p_{t}$, and also those generated ancestrally through the layers of the network i.e., $g_{t}\to p_{t}\to x_{t}$ and $g_{t-1}\to g_{t}\to p_{t}\to x_{t}$. When using memory in inference, $L_{p_{t}}$ includes a memory loss between the retrieved memory and the inferred $p_{t}$.

We use backpropagation through time truncated to 25 steps - this means we roll forward the inference network for 25 steps, collect the errors and then backpropagate. We then roll forward the inference network from where we left off etc - i.e., we do not use a sliding window. Longer BPTT lengths are useful as getting an early $p_{t}$ wrong will form a bad memory, which then influences the memory retrieval many timesteps into the future. We limit it to 25 for reasons of computational efficiency. We optimize with ADAM (Kingma and Ba, 2014) with a learning rate that is annealed from 1e−3 to 1e−4. Initially we down-weight costs not associated with prediction ($L_{g_{t}}$ and $L_{p_{t}}$). We do not train on vertices that the agent has not seen before. We use tensorflow (Abadi et al., 2016) to implement the model, and code is available at http://www.github.com/djcrw/generalising-structural-knowledge.

#### Simulation details

All the tasks described below are best ‘solved’ if the underlying structure is learned, even though each structure is different. We now describe the details for the types of graphs we considered, as well as the simulation details.

For all simulations presented above, we use the additional memory module (two separate memory matrices) in grid cell inference, but introduce it gradually during training via a weighting term to the variance of $q_{\varphi }\left(g_{t}|x_{\leq t},M_{t-1}\right)$. Each time the agent enters a new environment, both memory matrices, M, are reset (all weights zero). Asides from when otherwise stated, the agent randomly diffuses through each environment.

The agent is initially randomly placed in each environment. The agent changes to a completely new environment after a certain number of steps (∼ 2000-5000 for the 2D graph worlds, lower for smaller environments/ tasks). For 2D graph worlds, typically after 200−300 environments, the agent has fully learned the structure and how to address memories. This equates to ∼50000 gradient updates (1 gradient update per block of 25 steps). For smaller worlds, learning is much faster.

We now describe the dimensions of variables (summarized in Table S2). We use $n_{s}=45$ (the number of different sensory objects), $n_{s^{\text{∗}}}=10$ (the compressed sensory dimension) and 5 different scales/modules. The number of TEM entorhinal cells in each scale/module are [30,30,24,18,18], and the number of TEM entorhinal cells that project to TEM hippocampus, $n^{f}$ are [10,10,8,6,6] (i.e., the first 1/3 entorhinal cells in each scale/module). Thus the number of hippocampal cells in each scale/module are [100,100,80,60,60] i.e., $n_{s^{\text{∗}}}$ multiplied by each $n^{f}$. λ and η both start low and then rise to 0.9999 and 0.5 respectively during training.

As mentioned in Section ‘Task details’, for each task we train on environments of different sizes - this means a true abstract representation must be learned and not just one that is a template map. The learned map must generalize to different sized worlds.

We now describe additional simulation details specific to each task.

#### Transitive inference

When the agent navigates these line graph environments, the actions, a, given to TEM are two dimensional with the first element describing higher/lower and the second element by how much.

#### Social hierarchy

When the agent navigates these graph, the actions, a, given to TEM are a one-hot encoding of relations such as ‘child of’, ‘grand-parent of’, ‘sibling of’ etc. There are 10 available actions overall.

#### 2D graphs

We run simulations in either 6-connected graph worlds, or 4-connected graph worlds. The action is a one-hot encoding - either 4 or 6 dimensional depending on square or hexagonal worlds respectively.

For **diffusive behavior**, the agent has a slight bias for straight paths to facilitate exploration in these larger worlds. We show all TEM learned entorhinal cells in Figures S5A–S5D for a hexagonal and a square environment, and all hippocampal cells in Figure S5E for a hexagonal environemnt. We note that even in hexagonal worlds TEM sometimes learns hexagonal grid-like cells and sometimes square grid-like cells.

For **non-diffusive behavior** (e.g., simulations involving object vector cells), we bias the agents transition behaviors to head toward shiny objects (for object vector cells) or spend more time near boundaries (for border cell representations). For object-vector cell simulations, we also use an additional distribution in grid cell inference: $q_{\varphi }\left(g_{t}|s_{t}\right)$, where $s_{t}$ is an indicator saying whether the agents is at the location of a ‘shiny’ state. This means that the entorhinal cells can know when it is at a ‘shiny’ object state. From this, the network builds its own representation encoding vectors from the shiny states. We let this knowledge go to only a singe scale module for which we change $f_{g}\left(\ldots \right)$ to a leaky rectified linear unit additionally thresholded at 1. We make one further change to the generative network to encourage the learning of vector representations, by not telling the generative model what action, $a_{t}$, was taken. This encourages it to build representations of what actions will likely be taken (as governed by behavior). Interestingly, this phenomena is observed in sleep (generative) replay - sequences of head direction activations are divorced from replay of sequences of awake activity locations (Brandon et al., 2012).

#### Hexagons versus squares

In the main text we presented grid cells that have a hexagonal tiling pattern. However hexagons are not all we find. For example square grid cells can be found in both hexagonal and square worlds. Importantly periodic representations are **always** found.

We note that, like Cueva and Wei (2018) and (Banino et al. (2018), a higher ratio of grid to band cells is observed if regularisation of grid cell activity is used (encouraging the square of grid activity to be low). A recent theoretical paper (Sorscher et al. (2019) proved analytically that the key determinant to hexagon versus square is *how* the grid cell activity is regularised. Non-negativity constraints, or regularisation with a dominant 3rd order term enforce wavevector triplets leading to hexagonal grid cells.

#### Complex spatial tasks

We increase the backpropagation through time truncation to 100 so that gradient information has access to the whole state space. The number of TEM entorhinal cells in each scale/module are [18,18,15,15,15], and the number of TEM entorhinal cells that project to TEM hippocampus, $n^{f}$ are [6,6,5,4,4] (i.e., the first 1/3 entorhinal cells in each scale/module). Thus the number of hippocampal cells in each scale/module are [60,60,50,40,40] i.e., $n_{s^{\text{∗}}}$ multiplied by each $n^{f}$.

We show additional examples of cells that ‘chunk’ as well as those that don’t, from TEM’s hippocampal and entorhinal layers in Figures S5F and S5G.

#### Analysis of remapping data: Preserved place cell-grid cell relationships across environments despite remapping

##### Experimental prediction

Our theoretical framework predicts place cells and grid cells retain their relationships across environments - despite place cell remapping - to allow generalisation of structural knowledge encoded by grid cells. More specifically, our framework predicts the following to be true: 1) As has been previously observed experimentally (Fyhn et al., 2007), our framework predicts that when an animal is moved from one environment to a structurally similar environment but with different sensory experiences, place cells will undergo remapping (e.g., Figure 4E main text), and grid cells will realign (e.g., Figure 4A main text). 2) As has also been previously observed experimentally (Fyhn et al., 2007), we predict the grid cell correlation structure (i.e., relationships between grid cells) within a module will be preserved across environments. 3) Despite realignment and remapping, we predict that, within a grid module, a given place cell will retain its relationship with a given grid cell across environments. For example, if a given place cell’s firing field is in a given grid cell’s firing field in one environment, it should remap to a location in a second structurally similar environment that is also in a firing field of that grid cell (Figure 6 main text).

We empirically test for a preserved place cell-grid cell relationship across environments in two datasets from different remapping experiments, in which both grid and place cells were recorded across different environments. We first briefly describe the experimental setup of the experiments, followed by the details of the analyses and results that support our prediction in both datasets. We additionally demonstrate that these results cannot be explained by the place and grid cells not remapping or realigning, and, as has been previously shown (Fyhn et al., 2007), that the correlation structure of grid cells is preserved across environments.

##### Dataset 1 - Barry et al., 2012

In the first dataset (Barry et al., 2012) - dataset 1 - both place and grid cells were recorded from rats in two different environments. The environments were geometrically identical $1m^{2}$ arenas that were in distinct locations in the recording room, and differed in their sensory (texture/visual/olfactory) experiences. Each of seven rats had recordings from both environments in MEC and hippocampal CA1. Each recording day consisted of five twenty-minute trials, in which the rat free foraged in the environments. In between trials the rat was taken out of the arena. Of the five trials on a given day, trials 1 and 5 were in one environment, which the animal is familiar with (having spent at least 100 minutes in the environment), and trials 2-4 were exposures to a second, novel environment. We can therefore test for preserved place cell-grid cell relationships both within and across environments in this dataset.

Barry et al. (2012) sought to establish the effects of environmental novelty on grid and place cell properties, finding an increase in grid scale and decrease in grid score, as well as an increase in place cell field sizes in novel environments. This effect reduced with exposure to the novel environment over the course of trials 2-4, such that grid and place cells on trial 4 had properties most comparable to those on trials 1 and 5 (Barry et al., 2012). We therefore restrict our analyses of the second environment to trial 4. Further details about the experimental setup can be found in Barry et al. (2012).

##### Dataset 2 - Chen et al., 2018

We repeat our analyses in a second dataset (Chen et al., 2018) - dataset 2. In dataset 2, both place and grid cells were recorded as mice free foraged in both real and virtual reality environments. These real and virtual environments provide the two different environments for the across environment measures of place cell-grid cell relationships. We do not have a ‘within environment’ condition for this dataset. As described in full in Chen et al. (2018), in the virtual reality environment the mice were head-constrained such that head movements were constrained to rotations in the horizontal plane while the mouse runs on a Styrofoam ball. Screens and projectors projected a virtual environment around the mouse and onto the floor from a viewpoint that moves with the rotation of the ball. Hence this system allows expression of free foraging spatial navigation behavior, analogous to that in the real world.

Both the real and virtual reality environments were square, and size $60cm^{2}$. Trials in the real and virtual environments were 20 and 40 minutes long, respectively. Recordings were made in MEC and hippocampal CA1. (Chen et al., 2018) showed that spatial neuronal cell types that typically characterize 2-dimensional real space, including place cells and grid cells, could be measured in the virtual environment. Of the eleven mice that were trained in the virtual reality system, four had recordings from both place and grid cells, and could therefore be included in our analyses. Further details about the experimental setup and virtual reality system can be found in Chen et al. (2018).

Details of the number of cells recorded in each animal are found in Table S3.

### Quantification and Statistical Analysis

#### Data analyses to test for preserved place cell-grid cell relationship

We tested the prediction that a given place cell maintains its relationship with a given grid cell across environments using two measures. First, whether grid cell activity at the position of the peak place cell activity is correlated across environments (gridAtPlace), and second, whether the minimum distance between the peak place cell activity and a peak of grid cell activity is correlated across environments (minDist; normalized to the corresponding grid scale).

#### Data pre-processing and critical controls

In the tests presented later, we show results for raw data where we take several steps (with different strictness levels) to avoid possible confounds. Results are shown for all combinations of these choices in Table S4. These include:

#### Defining a grid-score cut-off to ensure entorhinal cells were grid cells

To ensure we are robust to the quality of grid cells entering the analysis, we consider several different grid score cut-offs. We use cut-offs of 0, 0.3 and 0.8. Using less stringent grid cut offs allows more cells and animals into the analysis (Table S3). We would expect our effect to be weaker when reducing the grid score cut off, as the resulting rate maps are likely to be less representative of the grid cell population. Both grid score and scale were computed as in Barry et al. (2012).

#### Fitting idealized grids to ensure grid-peaks were well-defined

We fit the recorded grid cell rate maps to an idealized grid cell formula (Equation 6 from Stemmler et al., 2015), and use this ideal grid rate map to give grid cell firing rates and locations of grid peaks (Figures S6A–S6C). This leads to a very strenuous control as it ensures that results cannot be driven by *any* differences across grid cells apart from grid phase, grid scale and grid angle (which are the only fitted parameters). This additionally allowed us to use grid peaks that were outside the box. We only fitted idealized grids in situations where we also defined a grid-score cut off (g = 0.8) to ensure good model fits.

#### Removing place cells at borders to ensure effects are not driven by border cells.

Here we removed all cells whose peaks were ≤10% of environment width from the border. The reason we wish to account for border effects is because non-grid MEC cells (such as border cells) rather than grid cells may drive place cell remapping to the borders. We have this criteria for all our analyses.

#### Ensuring cells have actually remapped

Though not data-preprocessing, we ensure that any results could not be confounded by place cells and/or grid cells not remapping/ realigning (i.e., the animal thinking it was still in the same box!). We test this by examining the distributions of spatial correlations obtained when correlating a given place or grid cell’s rate map in one environment with its rate map in a second visit to that same environment (within environments; only possible in dataset 1) or its rate map in a different environment (across environments). In dataset 1, we found that all the grid cells realigned across environments and the place cells remapped, with spatial correlation coefficients around 0 and distributions similar to those observed in hippocampal global remapping experiments (Fyhn et al., 2007) (Figures S6D and S6E). On the other hand, spatial correlations were high upon a second visit to the same environment. Distributions of spatial correlations near 0 for both place and grid cells across environments were also found in dataset 2 (Figures S6F and S6G). These results suggest that, as expected, grid cells realigned across the environments and the place cells accordingly underwent global remapping; global place cell remapping generally accompanies grid realignment (Fyhn et al., 2007). That the place and grid cell spatial correlations were near zero means it would be a non-trivial result should the place and grid cell relationship be preserved.

#### Computing the measures

We first perform the data-preprocessing, making each cell pass the appropriate checks.

We would like to know whether the relationship between place and grid cell pairs is preserved across environments. We propose 2 measures.

1) **Does a given grid cell fire similarly at the respective peaks of a given place cell in both environments?** We take a place cell and look at its peak in both environments, which we call P1 and P2. We then take a grid cell, and look at its firing rate at P1 in env1 - we call this X. We look at its firing rate in env2, we call that Y. This gives us a datapoint [X,Y]. We then do this again for the next grid cell, which gives another datapoint. We loop through all the grid cells and place cells for the same animal. Then start again for the next animal. We can then plot all these points on a graph, and find the correlation coefficient - this is the gridAtPlace measure (Figure S7A).

For the place cells, we analyzed cells defined as place cells in Barry et al. (2012) and Chen et al. (2018). Locations of place cell peaks were simply defined as the location of maximum activity in a given cell’s rate map.

We require each place-grid pair to come from the same animal, but we do not require that the place and grid cells were simultaneously recorded i.e., a place cell may be paired with a grid cell from a different recording session.

Note: If there were only a single grid frequency (or module) in entorhinal cortex, TEM would predict a near perfect correlation across environments between gridAtPlace scores for each grid-cell place-cell pair. However, if either (1) place cells are influenced by phases of more than a single grid module or (2) place cells predominantly received input from a single grid module, but we (the experimenter) do not know which module (as is the case), then we should not predict perfect correlations, only non-zero correlations.

#### Statistical testing

To test the significance of this correlation and ensure it is not driven by bias in the data, we generated a null distribution by permuting the place cell peak (5000 times) and recomputing the measures and their correlation across trials. We use two possible ways of permuting. First, we choose a position randomly (but still passing our pre-processing steps). Second we choose a position from another recorded cell (cells from same and other animals to get enough combinations). We then examine where the correlation coefficient of the non-shuffled data lies relative to the null correlation coefficients to determine its statistical significance. These analyses were carried out separately for both datasets. Again, results from both procedures (for all tests) are reported in Table S4.

#### Which cell types generalize their structure across environments?

As a brief interlude before the main result, we first test whether the correlation structure of each cell type generalizes across environments.

#### Grid cells realign and keep their correlation structure

Indeed, although grid cells realign across environments, their correlation structure is preserved (Fyhn et al., 2007). Although this has been previously demonstrated, we also showed it to be true by demonstrating that the correlation structure between the grid cells was itself correlated (i.e., preserved) across environments. More specifically, we calculated the grid cell by grid cell spatial correlation matrix in one environment, and correlated its upper triangle with that of the correlation matrix in the other environment (a correlation matrix of the same grid cells, but computed in a different environment). We tested this in the single animal with the most recorded grid cells across both environments in each dataset (in a rat with 15 grid cells in dataset 1 [comparing trials 1 and 4], and a mouse with 13 grid cells in dataset 2). This was significant relative to a null distribution generated by permuting grid cell-grid cell pair correlations in both dataset 1 (r = 0.55, p <0.001; Figure S7B) and dataset 2 (r = 0.95, p < 0.001; Figure S7D). These results are expected if the grid cells encode knowledge that generalizes across environments. A similar result has previously been reported in Yoon et al. (2013) which included in one of our datasets.

Place cells remap, only weakly retaining correlation structure across environments

We also found this effect to be weakly significant in place cells in dataset 1 (r = 0.31, p = 0.035; Figure S7C) and not significant in dataset 2 (r = 0.16, p = 0.21; Figure S7E).

#### Preserved relationship between grid and place cells across environments

Back to our main results in examining whether grid-cell place cell relationships are preserved across environments using our two measures (gridAtPlace and MinDist).

##### Dataset 1 - Barry et al., 2012

As a sanity check, we first confirmed these measures were significantly correlated within environments, i.e., correlated across two visits to the same environment (trials 1 and 5), when the cell populations have not remapped. We see that for both measures there is a significant correlation across the trials (the true correlation coefficient is above 95%of the null distribution of correlation coefficients; Figure S7F), for 445 place cell-grid cell pairs. This indicates that upon returning to the same environment, place cells and grid cells have retained their relationship with each other, as expected.

We then tested across environments, i.e., visits to two different environments (trials 1 and 4), to assess whether our predicted non-random remapping relationship between grid and place cells exists. Here we also find significant correlations for all combinations of measures, preprocessing decisions and statistical tests (Table S4). Data for the most stringent/conservative set of inclusion criteria (grid score > 0.8, leaving 115 cell pairs) are shown in (Figure S7G, gridAtPlace p<0.005, minDist p<0.05).

##### Dataset 2 - Chen et al., 2018

In this dataset, we only have measures for across environments, i.e., visits to the real and virtual worlds. We again found that the gridAtPlace measure was significant across all combinations of measures, preprocessing decisions and statistical tests (Table S4). Here the minDist measure is trending significance (but note this dataset has far fewer cell pairs (Table S4). Figure S7H shows data for the 64 pairs that survived the most stringent inclusion criteria (gridAtPlace p<0.05, minDist p=0.0524)

#### Remarks

Together, these are the first analyses demonstrating non-random place cell remapping based on neural activity, and provide evidence for a key prediction of our model: that place cells, despite their remapping and grid realignment across environments, retain their relationship with grid cells.

#### Analysis of lap-specificity

We followed Sun et al. (2020) in their definition of ESR cells. For each cell, we have t=15 trials each of n=4 laps of a l=8 loop. We 1) Average over trials to get a cell’s activity profile over 4 laps, 2) compute the spatial activity as the average across laps, 3) find the spatial bin of peak spatial activity, *s*, 4) subtract spatial activity from lap activity (for each lap) to give model-corrected (MC) activity and 5) get MC activity at location *s*. This gives a vector of 4 numbers which is the ESR activity.

Sun et al. (2020) used permutation testing to determine whether cells were ESR cells or not, but since we have no noise we use another measure. We define non-spatial cells to be those with peak ESR activity / peak spatial activity over 1.25.

#### Remapping in non-spatial task analysis

To determine ESR correlations, the ESR activity in one environment was correlated with the ESR activity in another. Similarly for spatial correlations we correlated spatial activity.

Because we only had, at most, 250 place cells in TEM for each environment, we considered 6 different environments and computed the ESR correlations and spatial correlations for each cell in each environment pair to increase the number of reliability of our distributions.

^3^$p_{t}^{x}$ is an intermediary variable retrieved via the memory $M_{t-1}$ from $x_{t}$—i.e., it represents $x_{\leq t}$ and $M_{t-1}$ in the posterior for <.
