# Supplementary material for: The Tolman-Eichenbaum Machine: Unifying Space and Relational Memory through Generalization in the Hippocampal Formation
Source: Cell. 2020 Nov 25;183(5):1249–1263.e23. doi: 10.1016/j.cell.2020.10.024 (PMC7707106; doi:10.1016/j.cell.2020.10.024)
Supplement: Document S1. Tables S1–S4 [file mmc1.pdf]

Cell, Volume *183*

Supplemental Information

The Tolman-Eichenbaum Machine: Unifying Space and Relational Memory through Generalization in the Hippocampal Formation

James C.R. Whittington, Timothy H. Muller, Shirley Mark, Guifen Chen, Caswell Barry, Neil Burgess, and Timothy E.J. Behrens

	Name	Function
**Gradient descent weights**	$\mathbf{W}_a$	Action dependent transition weights
	w_x	Scales sensory prediction
	w_p	Scales sensory input to HC
	α^f	Temporal filtering parameter
**Fixed weights**	$\mathbf{W}_{repeat}$	Repeat each element of vector in place a specified number of times
	$\mathbf{W}_{tile}$	Make copies of vector and concatenate
**Hebbian weights**	$\mathbf{M}$	Store memories, used in attractor network

Table S1: **The different types of weights in TEM, related to Figures 2 and STAR Methods.** We note that there will be other weights matrices in TEM where we have used a MLP function, though they simply serve as function approximators.

Variable	Stream 1	Stream 2	Stream 3	Stream 4	Stream 5	Total
Entorhinal, $\mathbf{g}$	30	30	24	18	18	120
Entorhinal down-sampled, $f_{down}(\mathbf{g})$	10	10	8	6	6	120
Hippocampus, $\mathbf{p}$	100	100	80	60	60	400
Temporally filtered sensorium, $\mathbf{x}^f$	10	10	10	10	10	50
Compressed sensory observation, $\mathbf{x}^c$	10	-	-	-	-	10
Sensory observation, $\mathbf{x}$	45	-	-	-	-	45

Table S2: **Table showing the number of neurons for each variable/layer, related to STAR Methods.** Note the hippocampal dimensions, per stream, are the multiplication of entorhinal and sensory inputs - coming from the outer product of the two representations.

	Animal number	Grid cells	Place cells	Place-grid pairs
**Grid score** ≥ 0.8				
**Dataset 1**	1	9	12	108
	6	1	5	5
	7	1	2	2
**Dataset 2**	1	5	8	40
	2	1	6	6
	4	3	6	18
**Grid score** ≥ 0.3				
**Dataset 1**	1	15	12	180
	2	3	10	30
	3	2	6	12
	6	3	5	15
	7	1	2	2
**Dataset 2**	1	10	8	80
	2	1	6	6
	3	2	4	8
	4	5	6	24
**Grid score** ≥ 0				
**Dataset 1**	1	15	12	180
	2	4	10	40
	3	2	6	12
	5	1	4	4
	6	3	5	15
	7	2	2	4
**Dataset 2**	1	13	8	104
	2	2	6	12
	3	2	4	8
	4	5	6	24

Table S3: **Numbers of animals and cells that enter the across environments analyses, related to Figure 6 and STAR Methods.** Table showing the total animal and cell numbers that survive the criteria and make it into the analyses where the grid score cut off is $\geq$ 0.8, $\geq$ 0.3 and $\geq$ 0 for both datasets. Note: although the animal numbers may be the same across the datasets, they are different animals.

Dataset	Grid cut-off	Ideal grid	Perm type	GAP r	GAP p	MD r	MD p
1	0.8	1	0	0.322	**0.0014**	0.170	**0.0334**
1	0.3	0	0	0.654	**0.0094**	-	
1	0	0	0	0.630	**0.0318**	-	
1	0.8	1	1	0.322	**0.0038**	0.170	**0.0352**
1	0.3	0	1	0.654	**0.0356**	-	
1	0	0	1	0.630	0.0776	-	
2	0.8	1	0	0.273	**0.0396**	0.204	0.0524
2	0.3	0	0	0.554	**0.0282**	-	
2	0	0	0	0.544	**0.0404**	-	
2	0.8	1	1	0.273	**0.0468**	0.204	0.058
2	0.3	0	1	0.554	**0.0340**	-	
2	0	0	1	0.544	**0.0468**	-	

Table S4: **Analysis results for different parameter settings, related to Figure 6 and STAR Methods.** Ideal grid 1/0 is whether we used a fitted ideal grid ratemap or not respectively. Permutation type 0/1 is whether we sampled a place cell peaks randomly, or from another recorded place cells. GAP/MD is gridAtPlace and MinDIst respectively. We show the r value and the significant p for both. Results below a 0.05 significance threshold are shown in bold. Our analysis results are robust to parameter settings, with the non-significant results also trending.
